# Movement and Dispersion Parameters Characterizing the Group Behavior of *Drosophila melanogaster* in Micro-Areas of an Observation Arena

**DOI:** 10.3390/ani15111515

**Published:** 2025-05-22

**Authors:** Nam Jung, Chunlei Xia, Yong-Hyeok Jang, Hye-Won Kim, Yun Doo Chung, Tae-Soo Chon

**Affiliations:** 1 Research Institute of Computer, Information and Communication, Pusan National University, Busan 46241, Republic of Korea; uchpracacia@gmail.com (N.J.); c.xia2009@gmail.com (C.X.); 2 Research and Development, Ecology and Future Research Institute, Busan 46228, Republic of Korea; janegsa1@gmail.com (Y.-H.J.); hw102800@daum.net (H.-W.K.); 3 Department of Electrical and Electronics Engineering, Pusan National University, Busan 46241, Republic of Korea; 4 Department of Life Science, University of Seoul, Seoul 02504, Republic of Korea; ydchung@uos.ac.kr

**Keywords:** group movement detection, computational behavior, spatial pattern, behavior profile, diel difference

## Abstract

In the present study, groups of 10 adult males from wild-type strain *Drosophila melanogaster* Canton-S and corresponding mutant *tab2^201Y^* were continuously observed using automatic digitization. Data based on instantaneous movement and cumulated movement positions were obtained for micro-areas providing space for resources (food and moisture) and for activity (edge area and intermediate area [between edge and center-diffusion areas]) within the observation arena for 24 h. The results confirmed the natural tendency of local aggregation among individuals within the observation arena (14 cm × 14 cm) at a relatively low density of ten individuals. For Canton-S, temporal cooccurring patterns among different parameters were observed as time progressed, with two primary patterns identified in the resource supply areas: single peak and double peaks. The single peak was observed with maximum speed and *I*-index, indicating minimum degree of isolated individuals from groups, during the transition from the photoperiod to the scotoperiod. The double peaks occurred before (mid-to-late photoperiod) and after (end of scotoperiod) the single peak, co-occurring temporally with a number of parameters including duration rates, stop number (total occurrence of pauses), stop time (total duration of pauses), mean crowding (MC), and social space index (SSI), indicating local aggregations for feeding in accordance with maximum durations in resource supply areas. Temporally cooccurring trends in parameters were also found with the stop number and SSI in micro-areas associated with activity, indicating that short pauses were needed to keep balance between attraction and repulsion between nearby individuals. Overall, the measured parameters varied depending on the micro-area, light phase, and strain. In particular, behavioral differences were observed for *tab2^201Y^*, including an increase in speed, especially in the areas related to activity during the scotoperiod. Between strains, behavioral differences in the measured parameters were observed less for *tab2^201Y^* than Canton-S.

## Simple Summary

Studying local grouping behavior is essential for understanding animals’ social strategies and interactions related to survival and reproduction. It also provides insights applicable to ecological conservation and biotechnological innovation. In this study, after digitizing the continuous movements of *Drosophila melanogaster* for a whole day, we examined whether local grouping formed even at a relatively low density. Not only basic parameters such as linear and turning speeds, but also the multi-parameters covering instantaneous movement and dispersion patterns were recorded simultaneously across different micro-areas for resource supply and activity to comprehensively illustrate group behaviors. The parameters appeared in two patterns in the wild strain: one characterized by maximum speed and minimal separation of outliers from groups during the transition from light to darkness, and the other with many parameters showing local aggregation in the resource-supply (food and moisture) area before and after the maximum speed. Interestingly, all these parameters were significantly altered in a mutant strain, suggesting that genes contribute to local grouping behavior. In summary, our group behavior study contributes to unravelling the tendency of *Drosophila* to form groups, based on multi-parameter estimation observed across different micro-areas.

## 1. Introduction

The group behavior of individuals showing local enhancement in space has received significant research attention because it can be used to investigate survival fitness relating to sociality at the population level. Congregation in close proximity is the natural tendency of aggregated or social species for local enhancement as an adaptive behavior [1,2]. This has motivated group behavior research into individual interactions related to the specific behavioral intentions of the target species, which are usually associated with visual and olfactory cues [3,4,5] and/or their overall spatial conformation in local grouping. Although they are not eusocial insects, group behavior involving social mechanisms has been widely observed among members of the genus *Drosophila* [6,7], making them a suitable target for understanding the origin of social behavior.

Numerous accounts of specific interactions, which are usually associated with visual and olfactory cues [3,4], have been reported for *Drosophila*, including aggression with a focus on agonistic interaction processes [5,8,9,10], maintaining social status when interacting with other individuals [11,12,13], and identifying genetic [12,14,15,16] or physiological [4,17,18,19] mechanisms associated with aggression. Studies on positive individual interactions have been also reported, including courtship [20,21,22,23], cooperative search and defense [1,24,25,26], and aggregation [6,27,28,29]. In some studies, both negative and positive interaction behaviors have been investigated together [30,31,32], including field studies [33].

Research on spatial conformation has focused on investigating group formation mechanisms originated from local enhancement [1,2,34], mainly due to chemo-sensory contact cues [2,6,28,35,36]. Motivation for spatial conformation can also be divided into collective behavior and social networking [6]. Collective behavior is focused on overall group formation in space while social networks are centered around the functional properties of individual–group relationships. Because the relationship of a specific individual with the group is the focus of network analysis, the accurate tracking of the behavior of individuals is vital for the analysis of social networks, while individual monitoring is less crucial for the understanding of collective behavior [35].

In a study focusing on social networking, Simon and Dickinson [37] quantified the social interaction networks (SINs) of *D. melanogaster* by developing a behavioral classifier that identified when pairs of flies were within two body lengths of one another as either an interactor or interacted. Similarly, Schneider et al. [28] examined the sensory modalities that affect inter-fly interactions and SINs, reporting that the formation of nonrandom SINs depends on chemosensory cues. Social clustering has been observed to be a highly dynamic process that includes all individuals that participate in stochastic pair-wise encounters mediated by appendage touches [36]. The emergence of social clustering from group behaviors has also been comprehensively studied [38].

Compared with the network approach, the study of collective behavior mainly focuses on spatial group arrangements. For example, Sexton and Stalker [39] photographed the spacing of *Drosophila parmelanica* and reported a uniform spacing of 5 mm at maximum density, while Navarro and del Solar [40] provided evidence for gregarious behavior in *Drosophila* that was independent of sex and temperature in the observation arena. In addition, using automated devices and physiological experiments for the analysis of group behavior, Branson et al. [41] reported that the relative positions of flies during social interactions varied according to gender, genotype, and the social environment. Simon et al. [2] characterized a simple, resource-independent form of local enhancement, reporting that social space in *D. melanogaster* is within two body lengths and suggesting that this social space does not require the perception of the identified aggregation pheromone. Jiang et al. [36] also reported the emergence of a social cluster from collective pairwise encounters in *Drosophila* mediated by appendage touches and specific ppk neurons activated by contact-dependent social grouping.

However, due to the limitations associated with the continuous observation of multiple individuals, few studies have investigated collective behaviors continuously over long periods of time. Therefore, in the present study, we were motivated to investigate the continuous collective behavior of wild-type and mutant strains of *D. melanogaster*. The mutant *tab2^201Y^* strain has been widely used as a GAL4 driver for the mushroom body, which is a brain center for complex behaviors including learning and memory [42,43]. Numerous studies have used the *tab2^201Y^* strain to induce the expression of specific target genes under the control of an upstream activating sequence (UAS) in the mushroom body and investigated the effect of gene induction in fly behavior. Here, we also initially tried to use *tab2^201Y^* as a reference strain to study group behavior, but we found significant differences between *tab2^201Y^* and the wild-type control.

Hypothesizing that local enhancement could be objectively characterized according to multi-parameter measurements across different micro-areas through continuous observation, the following procedures were conducted in this study: (1) wild-type and mutant *D. melanogaster* were selected for comparison of group behavior, (2) the collective behavior of multiple individuals was continuously observed in different micro-areas, in relation to resource provision and activity within the observation arena, (3) the movements were digitized during different light phases over 24 h in an observation arena, (4) the observed data for group behaviors were quantified using a diverse range of movement parameters associated with instantaneous movement and cumulated movement positions, and (5) the patterns of multiple parameters were characterized by behavior profiling and temporal co-occurrences and compared between the two strains.

## 2. Materials and Methods

### 2.1. Rearing and Observation

**Figure 1:**
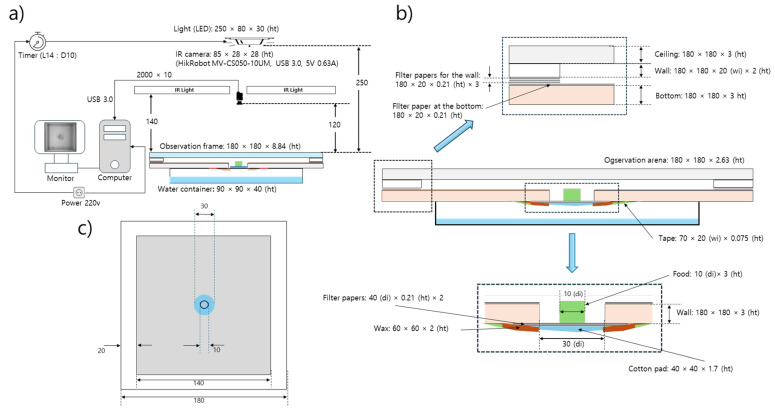
Observation system for detecting movement of *Drosophila melanogaster* (unit; mm). (**a**) Observation setup for continuous group movement, (**b**) closeup of observation frame (side view) and (**c**) observation arena (top view).

Wild-type *D. melanogaster* strain Canton-S and mutant *tab2^201Y^* were selected for the observation of group behavior in the present study. Ten adult males from each strain were continuously observed 3–5 days after emergence for 24 h across different light phases in the laboratory at a temperature of 24.1 ± 2.6 °C and a humidity of 52.5 ± 8.3% in an observation arena. The observation system was devised to continuously record group movements for the entire observational period and consisted of food and moisture supplies, lights, a camera, and a computer (Figure 1a,b).

The observation arena (140 mm × 140 mm × 2.63 mm) was in a polyethylene container surrounded by walls of 20 mm in width 2 mm in height (Figure 1b,c). In previous experiments, to prevent flies from walking on the side or the ceiling within an observation arena, chemicals have been applied to the walls [34,36] or the wings of the flies have been cut with surgical scissors [44]. However, in the present study, the flies were allowed to move freely within the observation arena, with no chemical or surgical treatments employed. While vertical test chambers have also been used to promote grouping [2], we used a horizontally oriented arena because similar local enhancement has been observed in horizontal chambers and both negative geotaxis and walking stress against gravity could be minimized [2,34]

In the middle of the observation arena, a hole 30 mm in diameter was cut to provide food for the test individuals (Figure 1b). Sugar (4%) mixed with agar [45] was used as the food source. Instead of using 1% agar as in similar experiments [36,46], we used a higher agar concentration (4%) to ensure sufficient rigidity for the entire 24 h observation period. Immediately before the observation, the solid agar-based food was cut into pieces with a diameter of 1 cm and a height of 3 mm and placed on a piece of cotton pad (40 mm × 40 mm × 1.7 mm) in the middle of the food-provision area (Figure 1b). The cotton pad was attached to the bottom of the observation arena and served as a stage supporting the agar food and providing moisture to the observation arena via the evaporation of water from a water container (90 mm × 90 mm × 40 mm; 10 mL of dechlorinated tap water) placed underneath the observation arena (Figure 1b).

Conditions of 14 h L–10 h D light were established using a white LED (12 V, 1.5 A; 780 lux) in the observation room for examining diel difference in movement behavior during the whole day observation. The light source was placed 250 mm above the observation arena, without potential impact of heat dissipation to the test individuals due to the light (Figure 1a). The light intensity was measured beside the observation arena at the same height. Additionally, an infrared light (850 nm; 12 V, 1.5 A) was placed 140 mm above the observation arena and used to detect individuals (Figure 1a).

In order to avoid compounding effects between the transplantation of the test individuals and changes in the light phase and to provide sufficient recovery time from cold anesthesia, the test individuals were introduced to the observation arena for at least 45 min during the scotoperiod before the start of photoperiod. All test individuals in each group cohabited in the same stock vial. Individuals were cold anesthetized before observation. To secure maximum time for handling individuals for observation while minimizing immediate cold stress, a stock bottle (glass; 93 mm × 23 mm (diameter)) with 40~50 individuals placed within it was placed inside a freezer. The stock bottle was taken out of the freezer after approximately 13 min, with the temperature within the stock bottle at −6.3 ± 3.6 °C. It took about 6.5 min to reach 0 °C within the stock bottle inside the freezer. Ten males were selected and introduced to the edge of the observation arena during the scotoperiod for acclimatization at least 45 min before starting observation in the photoperiod, as stated above. Observation was conducted continuously for 24 h during the photoperiod (14 h) followed by the scotoperiod (10 h).

### 2.2. Detection and Parameter Extraction

The individuals were observed using a 5 M pixel microscopic video (MV-CS050-10UM, USB 3.0, 5 V, 0.63 A, HikRobot®, Hangzhou, Zhejiang, China) at 15.88 frames per second (fps). The movement of the individuals was monitored using the convolutional neural network YOLOv8 (using python package: ultralytics ver.8.2.18) [47]. Individuals in groups were detected using multiple feature tracking including a Kalman filter and Hungarian assignment [31]. A time unit of 1 s was used to calculate the parameters from digital images. Time intervals of less than 1 s (e.g., 0.25 s) have been used in previous research to monitor the whole-body movement of *D. melanogaster* in response to external stimuli (e.g., toxins) [48,49,50]. However, because the overall positioning of the individuals was the main focus of this study, rather than the interaction of individual bodies or partial body movement, measuring the parameters at a time interval of 1 s was sufficient to present the overall position of multiple individuals over the entire 24 h period. This choice of time unit also reduced the computational time.

By consulting previous studies that employed various movement parameters when observing *Drosophila* behavior [44,48,50], we opted to consider two groups of parameters: those associated with instantaneous movement and those related to the dispersion of group movement positions cumulated over a certain period. Motility, sessility, and curvature of movement were selected as the instantaneous movement parameters. For motility, the speed, locomotory rate, and direction change rate (DCR) were measured. Speed was the mean of the measured values for all time units, while the locomotory rate was defined as the mean speed only when the individuals moved, excluding periods without movement. The DCR was defined as the angle change (without considering direction) after one time unit (1 s). To assess sessility, the stop number and stop time were measured during the observation period. A stop was defined as no movement (i.e., pause) within the time unit. The stop number was obtained by counting the number of initiated and terminated continuous pause for each light phase (4 h), while the stop time was the total duration of pauses in seconds within the light phase. To investigate the curvature of the movement tracks, the sinuosity was measured, comparing an individual’s actual track to the linear distance between its starting and ending points with the time *t* = 0, 1, 2, …, *T*, as follows [51]:

(1) $$\begin{array}{l} S=\frac{\sum_{t=0}^{T-1}dX_{t,t+1}}{dX_{0,T}},(t=0,1,2,\ldots ,T)\\ \Delta X_{i,j}=\sqrt{{\left(x_{i}-x_{j}\right)}^{2}+{\left(y_{i}-y_{j}\right)}^{2}} \end{array}$$

where Δ*X_i,j_* represents the displacement from time *i* to time *j*.

In addition to the instantaneous movement parameters, dispersion parameters were determined based on the cumulated movement positions within a fixed period. To describe group movements, Schneider et al. [28] proposed parameters associated with networking in group behavior, including the clustering coefficient, assortativity, betweenness centrality, and global efficiency. These parameters are useful for describing individual contact in social networks based on individual identification. In this study, we required parameters that were suitable for assessing collective behavior in terms of the overall spatial conformations without the need for individual identification during the observation period. We thus selected four parameters describing dispersion patterns related to cumulated movement positions: the number of clusters, the *I*-index, mean crowding (MC), and the social space index (SSI).

The number of clusters was selected to represent local group numbers created by local enhancement within the observation arena and was obtained using density-based spatial clustering (DBSCAN) [52]. The *I*-index was originally developed to measure the degree of spatial aggregation [53,54] and was determined as follows:

(2) $$I=\frac{(N+1)\sum_{i=1}^{N}r_{i}^{4}}{{\left(\sum_{i=1}^{N}r_{i}^{2}\right)}^{2}},$$

where *N* represents the total number of positions and *r_i_* is the distance to the nearest point from position *i*. In this study, we employed the *I*-index to represent the spatial isolation of individuals. Based on the equation for the *I*-index, isolated individuals have a greater distance to their nearest neighbor and the square of the sum of the squared distance (the denominator) increases faster than the sum of the double square of the nearest distance (the numerator). Consequently, if individuals are located far from other individuals that are closely grouped, the index decreases toward 0.

MC, calculated based on the average number of individuals in a unit area, was selected to represent the local crowdedness of individuals in a specified spatial unit [55,56]. MC (*c*) was calculated as follows:

(3) $$c≅m+\frac{v}{m},$$

where *m* and *v* are the mean and variance of the position densities in a spatial unit.

The SSI was selected to represent the balance between attraction and repulsion observed in nearby individuals, based on histogram representations of social distance [2,28,57]. The SSI was the percentage of flies in the first bin minus the percentage of flies in the second bin (SSI = first bin − second bin). An SSI equal to or lower than 0 suggests a lack of social interactions [2,28,34]. In this study, we used the frequencies of the first and second bins directly instead of normalizing using percentages to compare the effects of grouping between different micro-areas.

Dispersion parameters were tested across different spatial and time units to obtain optimal measurements. The closest distance between individual *D. melanogaster* has been reported to be less than 5 mm [2,39]. A slightly shorter distance of 4 mm was selected as the basis for determining the unit distance for the SSI and DBSCAN. For MC, the size of the observation arena was divided into different scales (1/4, 1/6, 1/14, 1/28, and 1/42) with reference to the length of the observation arena (14 cm). The dispersion parameters were measured using time windows from 10 to 60 s at 10 s intervals to determine the most suitable period for analyzing the dispersion patterns of cumulated movement positions. The values were also summarized for different light phases, with the data for the entire 24 h period split into six periods of 4 h each: three periods during the photoperiod (PI, PII, and PIII), the transitional period between the photoperiod and scotoperiod (P-S), and two periods during the scotoperiod (SI and SII).

### 2.3. Parameter Measurements in Micro-Area

We hypothesized that behaviors would differ between micro-areas within the observation arena. The arena was divided into four micro-areas: food provision, center diffusion, intermediate, and edge. Except for the food-provision area, the other three areas were defined according to DBSCAN (Version 1.4.2 provided in scikit-learn) using the cumulated movement positions within the observation arena.

### 2.4. Statistical Analysis

The parameters obtained in this study were generally skewed rightward (i.e., extremely high frequencies of low values); thus, they did not follow a Gaussian function. To obtain representative values for group behavior, we calculated the means of the parameters for each trial (eight in total for each strain). The mean and standard deviation (SD) for the trials were then employed as the representative values for the parameters, according to the micro-area, light phase, and strain, under the assumption that the means of the samples would follow a Gaussian distribution according to the central limit theorem [58,59]. The SD was used to evaluate the variability of the parameters.

It addition to their variability, the behavioral data in this study had an additional structural property regarding measurement dependence. Since observations were continuously conducted across micro-areas in the observation arena through the whole observation period, two factors, light phases in time and micro-areas in space, were dependent and coupled with each other. To examine the effects of these two factors on the coupled dependence, we conducted two-way repeated measurement ANOVA (SPSS ver.30.0.0.0 (172)) when considering the effects of the treatments on the observed data.

In this study, the practical research motivation was to investigate how group behaviors were differentiated, specifically among different levels of treatments in micro-areas (e.g., food provision, center diffusion) and separately, among light phases (e.g., PI, PII). For instance, during each light phase, we aimed to determine how the parameters (e.g., speed) were differentiated in each combination of micro-areas (e.g., food-provision area vs. center-diffusion area) and similarly, how the parameters were differentiated in each combination of light phases (e.g., PI vs. PII) in each micro-area. Multiple comparison tests would be suitable for this purpose. Multi-comparison in coupled dependence, however, was practically infeasible for comparing the effects of all combinations of treatments of two factors with coupled dependence (e.g., ‘Food-provision area during PI vs. Food-provision area during PII’, ‘Edge area during SI vs. Edge area during SII’), which totaled 24 combinations (= micro-areas (4) × light phases (6)). In addition to the large number of combinations, separate comparisons within each factor were not possible since the two factors were coupled.

Considering this difficulty of coupled dependence in multiple comparisons, we separated the data for micro-areas and light phases, releasing the condition of coupled dependence. For analyzing multiple comparisons among micro-areas, the data were separated according to light phases, while data for the four micro-areas in each light phase were dependent. Similarly, the data were separated according to micro-areas, while data for the six light phases in each micro-area were dependent. Before conducting multiple comparisons, the Friedman test (SPSS ver.30.0.0.0 (172)) was performed to check statistical differences in the treatments for each factor. The Friedman test is applicable to nonparametric repeated rank data for determining significance among treatments within a single factor. After checking significance across the total treatments, multiple comparison tests were subsequently conducted. To secure the differences in the highly variable parameter data, two tests were opted: the Wilcoxon signed-rank test [60] applicable to nonparametric data (rank) and the paired permutation test [61] applicable to parametric data (mean). Tests were conducted for each pair of treatments in each factor (e.g., 6 tests for micro-areas). To secure conservative significance, probabilities for alpha errors were decreased by applying additional degrees of freedom due to the number of paired tests as stated above; the original probabilities of alpha errors obtained from the Wilcoxon signed-rank and the paired permutation tests were divided by the number of paired tests for multiple comparison within each factor (6 for micro-areas and 15 for light phases). The final probabilities for determining alpha errors for significance were 0.0500/6 = 0.0083 for micro-areas and 0.05/15 = 0.00333 for light phases. To differentiate parameters between the two strains in each combination of micro-area and light phase, we also used the Wilcoxon signed-rank and paired permutation tests, which are applicable for comparing pairs.

## 3. Results

### 3.1. Overall Movement Positions and Parameter Frequencies

**Figure 2:**
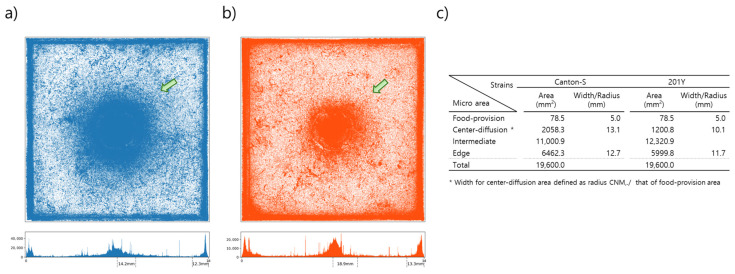
Cumulated movement positions of *D. melanogaster* and micro-areas within the observation arena. (**a**) Spatial positions for Canton-S (**b**) and *tab2^201Y^*, and (**c**) micro-areas according to spatial clustering.

Figure 2 presents the cumulated movement positions within the observation arena for 10 *D. melanogaster* adult males from strains Canton-S and *tab2^201Y^* over the 24 h observation period for all eight trials. The center area had a high density of movement positions for both strains (green arrows, Figure 2). It was observed that the center area with high densities was broader for Canton-S than in *tab2^201Y^*. The spatial clustering based on the accumulated movement positions was obtained from DBSCAN. The positions along the four sides of the observation arena were combined into one coordinate and clustering was conducted in one dimension. The center area with high cumulated positions was defined as the center-diffusion area after clustering, but the food-provision area (10 mm in diameter) within the center-diffusion area was excluded and instead considered separately as its own micro-area. The food-provision area was thus fixed at 78.5 mm^2^ for both strains, while the center-diffusion area was broader for Canton-S (2058.3 mm^2^) than for *tab2^201Y^* (1200.8 mm^2^) (Figure 2c). The edge area was similar for the two strains (6462.3 mm^2^ and 5999.8 mm^2^, respectively, for Canton-S and *tab2^201Y^*). The intermediate area was defined as the area between the center-diffusion and edge areas (Figure 2c) and was used to observe the activity of individuals in open space. The intermediate area was broader for *tab2^201Y^* (12,320.9 mm^2^) than for Canton-S (11,000.9 mm^2^).

**Figure 3:**
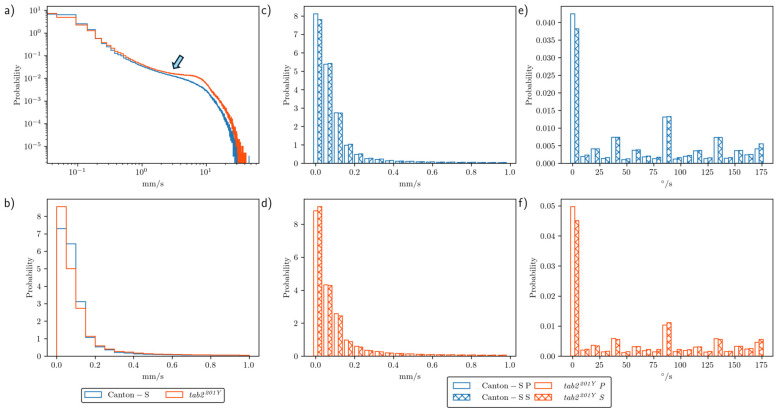
Speed and DCR for group movement for Canton-S and *tab2^201Y^ D. melanogaster* in two strains. (**a**) Log–log graph for speed frequencies (blue, Canton-S; orange, *tab2^201Y^*), (**b**) histogram for short range speed, (**c**) histogram comparing speed in photo- and scotoperiods for Canton-S and (**d**) for *tab2^201Y^*, and (**e**) histogram comparing DCR in photo- and scotoperiods for Canton-S and (**f**) for *tab2^201Y^*.

To assess the motility of group movement during the observation period, histograms for speed and DCR were obtained during the photo- and scotoperiods. Figure 3a presents the frequencies for speed over the entire observation period for Canton-S and *tab2^201Y^* on a log–log scale. Higher speeds were more frequent for *tab2^201Y^* than for Canton-S, especially above 1 mm/s (arrow, Figure 3a). Figure 3b displays the frequencies for speeds lower than 1 mm/s. These frequencies were highly skewed right, with extremely low frequencies above 0.2 mm/s.

Figure 3c,d compare the frequencies for speeds lower than 1.0 mm/s during the photo- and scotoperiods for Canton-S and *tab2^201Y^*, respectively. Frequencies were highly skewed right with extremely high frequencies of low speeds under 0.2 mm/s. The frequencies for speed during both the photo- and scotoperiods were similar overall for both strains (Figure 3c,d).

The frequency curves for the DCR were similar for the photo- and scotoperiods, with a low range for both strains (Figure 3e,f). The first bin (0–9°/s) had a very high frequency, indicating a straight and forward direction of group movement. The DCR was also higher for the angles close to 90°/s and 180°/s for both strains, while the frequencies for movement at angles of multiples values of 25°/s (e.g., 45°/s and 70°/s) were relatively higher than other angles for both strains (Figure 3e,f).

**Figure 4:**
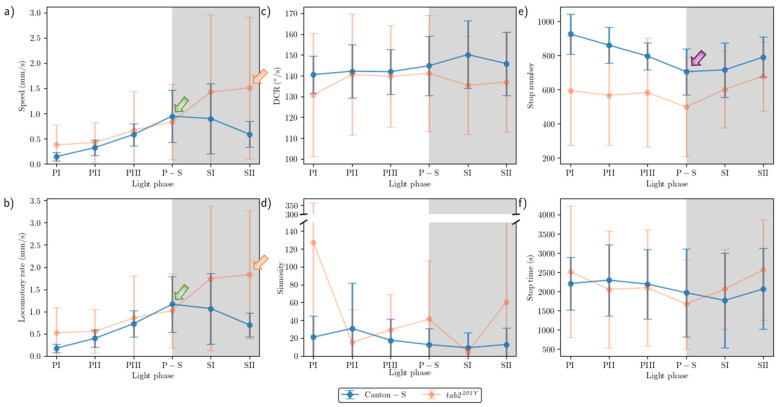
Movement parameters in the observation arena across light phases for Canton-S and *tab2^201Y^* of *D. melanogaster*. (**a**) Speed, (**b**) locomotory rate, (**c**) DCR, (**d**) sinuosity, (**e**) stop number, and (**f**) stop time.

The movement parameters for the two strains within the observation arena according to the light phase are presented in Figure 4. The diel difference within the observation arena differed between the two strains. For Canton-S, a peak in speed (1.0 mm/s) was observed during P-S (i.e., the transitional period between the photoperiod and the scotoperiod; green arrow, Figure 4a) while for *tab2^201Y^*, the speed continuously increased until the end of the scotoperiod (1.5 mm/s) (orange arrow, Figure 4a). Speed was higher overall for *tab2^201Y^* (0.9 mm/s on average) than for Canton-S (0.6 mm/s on average), particularly during the scotoperiod (1.5 mm/s and 0.7 mm/s on average, respectively). During the photoperiod, the speed of *tab2^201Y^* (0.5 mm/s) was slightly higher than that of Canton-S (0.4 mm/s), indicating that the higher overall speed for *tab2^201Y^* (Figure 3a) was mainly due to activity during the scotoperiod.

The locomotory rate was also measured to assess the motility of groups during only those time units when the individuals moved (Figure 4b). The locomotory rates were overall close to the speed for both Canton-S and *tab2^201Y^*. A slight increase was observed in the locomotory rates compared to the speed across the light phases, with the maximum observed during P-S and SII for Canton-S (1.2 mm/s) and *tab2^201Y^* (1.8 mm/s), respectively. No qualitative difference between the speed and locomotory rate was observed, because only a small number of stops occurred during the group movement of *D. melanogaster* within the observation arena during the observation period.

In contrast to the speed, the DCR (Figure 4c) was stable across the light phases at around 140.9°/s without a clear diel difference between the two strains (Canton-S: 144.2°/s; *tab2^201Y^*: 137.4°/s), with slight differences including a slight increase for Canton-S (145.8°/s) and a slight decrease for *tab2^201Y^* (136.8°/s) during SII.

Sinuosity (Figure 4d) was stable overall at an average of 17.3 across the light phases for Canton-S, though it rose to 23.1 and decreased to 10.9 during the photoperiod and scotoperiod, respectively. For *tab2^201Y^*, sinuosity was particularly high during PI (127.7) and SII (60.3). Except for these periods, however, the sinuosity had a stable range of 22.4 on average (Figure 4d).

The stop number exhibited an opposite pattern to the speed and locomotory rates, with a minimum (704.8) during P-S for Canton-S (purple arrow, Figure 4e), indicating that the stop number decreased when the speed increased. The stop number was consistently lower for *tab2^201Y^* than for Canton-S during all of the light phases (Figure 4e). The diel differences for *tab2^201Y^* during the photoperiod (581.2) and scotoperiod (639.7) were not as large as for Canton-S, showing 860.7 and 753.1, respectively. Like Canton-S, the minimum stop number for *tab2^201Y^* was observed during P-S (498.5).

The pattern for the stop time according to the light phase was markedly different from that for the stop number (Figure 4f). The stop time was relatively stable without a clear diel difference, reaching 2229.4 s during the photoperiod and 2915.3 s during the scotoperiod on average for Canton-S. The trend in the stop time was also stable, covering a narrow range for *tab2^201Y^* during the photoperiod and scotoperiod (2220.7 s and 2309.8 s, respectively).

The SD (vertical bars in Figure 4) varied greatly according to the light phase and strain. For Canton-S, the SD range was shorter overall and more stable across the light phases than for *tab2^201Y^*. Regarding the DCR, the SD was consistently high across the light phases for *tab2^201Y^* compared with Canton-S. For *tab2^201Y^*, SDs for the stop number and time were relatively high during the photoperiod and those for the speed and locomotory rate were relatively high during the scotoperiod (Figure 4). High SDs for sinuosity at PI, P-S, and SII were also noted for *tab2^201Y^*.

### 3.2. Duration Rates for the Micro-Areas

**Figure 5:**
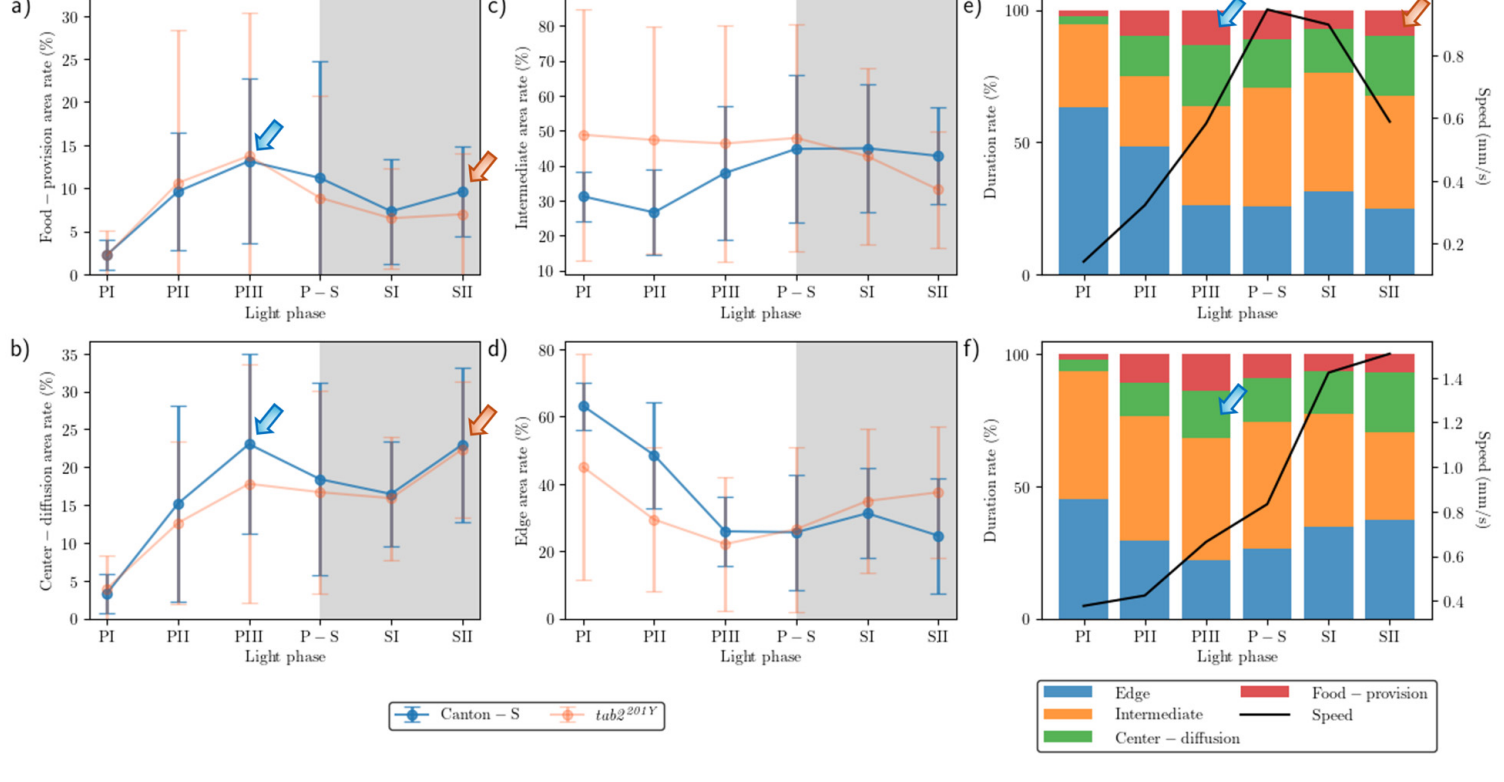
Duration percentages at micro-areas and behavior profiles for Canton-S and *tab2^201Y^ D. melanogaster*. (**a**) Duration (%) staying in the food-provision, (**b**) center-diffusion, (**c**) intermediate, and (**d**) edge areas within the observation arena, and (**e**) duration (%) superimposed with speed for Canton-S and (**f**) for *tab2^201Y^*.

The duration rate varied between the micro-areas (Figure 5). In the food-provision and center-diffusion areas, the trend in the duration rate was similar across the light phases and between the two strains, with two peaks observed for both strains during the mid-photoperiod (13.1–14.7% and 17.8–23.0% for the food-provision and center-diffusion areas, respectively) (blue arrows, Figure 5a,b) and late scotoperiod (7.0–10.0% and 22.3–23.0%, respectively) (orange arrows, Figure 5a,b). The duration rates in the center-diffusion area were higher (13.9% during the photoperiod and 19.7% during the scotoperiod on average) than in the food-provision area (8.3% and 8.5% on average, respectively).

The duration rate for the areas related to activity (Figure 5c,d) was substantially different from those areas that provided resources (Figure 5a,b). For Canton-S, the duration rate in the intermediate area was low during the early photoperiod (31.2%), increased during P-S, and stayed at a similar level during the scotoperiod (42.8%) (Figure 5c). In the edge area, the duration rate for Canton-S was substantially different from the other micro-areas, being initially very high at 63.2% during PI, decreasing rapidly until PIII, and then staying at a stable level around 26.9% (Figure 5d).

While the duration rate according to the light phase was similar between Canton-S and *tab2^201Y^* in the resource supply areas, changes between the strains were observed in the areas related to activity (Figure 5c,d). In the intermediate area, the duration rate for *tab2^201Y^* was higher during the photoperiod than for Canton-S, with 47.5% and 31.9% on average, respectively, and lower during the scotoperiod, with 37.9% and 43.9% on average, respectively (Figure 5c). Some differences in the duration rate were also observed in the edge area, being lower during the photoperiod (32.2% on average) and higher during the scotoperiod (36.2% on average) for *tab2^201Y^* than for Canton-S (45.9% and 28.0% on average, respectively) (Figure 5d).

As with the movement parameters, high variability in the SD was also observed for the duration rate. The SD range was higher for *tab2^201Y^* than for Canton-S, with a wide range during the photoperiod in the intermediate area and during PII–PIII in the food-provision and center-diffusion areas (Figure 5a–d). For Canton-S, the SD range was generally limited except during PII–PIII in the food-provision and center-diffusion areas.

Group behavior profiles are illustrated in Figure 5e,f, indicating the speed and duration rate in combination according to the light phase for the two strains. For Canton-S, the speed reached its peak (1.0 mm/s) during P-S. Before P-S, a high duration rate in the food-provision area was observed during PIII (13.1%) (blue arrow, Figure 5e), a consequence of the high speed during P-S after staying in the food-provision area during PIII. The speed continuously decreased after this until SII (0.6 mm/s), although the duration rate in the food-provision area increased again (9.6%) (orange arrow, Figure 5e).

Different profiles for the speed and duration rates were observed for *tab2^201Y^*. During PIII, the duration rate in the center-diffusion area was high (17.8%) (blue arrow, Figure 5f). However, unlike Canton-S, the speed continuously increased to reach its maximum (1.5 mm/s) during SII.

### 3.3. Dispersion Parameter Measurements

**Figure A1:**
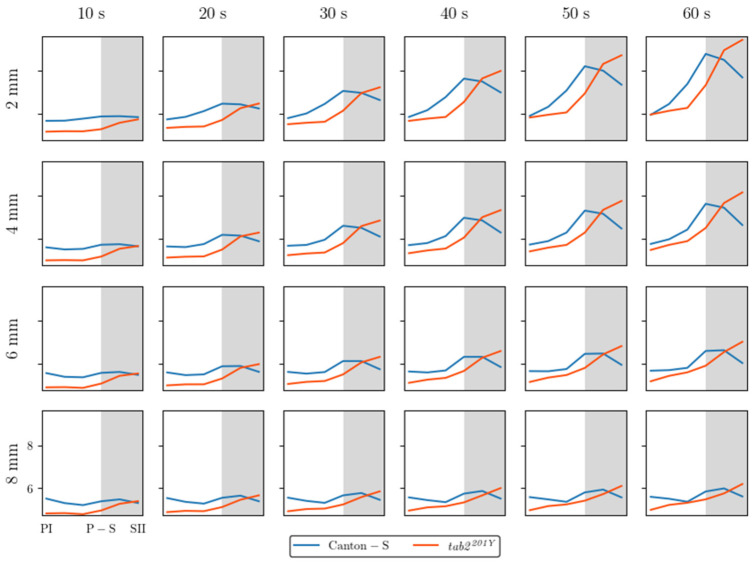
Cluster numbers in cumulated movement positions according to DBSCAN across different levels of threshold distance (ε) and time window sizes with normalization for Canton-S and *tab2^201Y^ D. melanogaster*.

Spatial and temporal units were determined to obtain dispersion parameters. Spatial clustering represents how many local groups were observed in the cumulated group movement positions. In determining the spatial units, the threshold distance (*ε*) for clustering was examined from 2 mm to 8 mm at intervals of 2 mm across time window sizes from 10 s to 60 s at intervals of 10 s. The cluster numbers obtained using the different spatial units and time windows according to the light phase are listed in Figure A1. The number of clusters increased as the window size increased, while the trend in cluster numbers according to the spatial unit size was similar overall, though there was a slight difference between the two strains during the late scotoperiod.

**Figure 6:**
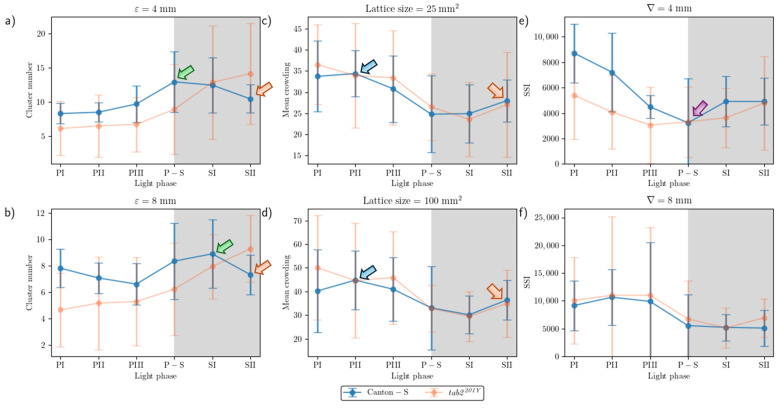
Dispersion parameters of group movement positions for Canton-S and *tab2^201Y^ D. melanogaster*. (**a**) Cluster numbers with *ε* equal to 4 mm and (**b**) 8 mm, (**c**) MC with unit size equal to 25 mm^2^ and (**d**) 100 mm^2^, and (**e**) SSI with threshold distance (*l*) equal to 4 mm and (**f**) 8 mm.

To examine the cluster patterns in more detail, we selected *ε* = 4 mm and 8 mm as the threshold distance, with 30 s as the window size. The trend over time for the cluster number with the two threshold distances was generally similar (Figure 6a,b). With *ε* = 8 mm, the cluster number (4.7–9.3 on average) was lower than *ε* = 4 mm (6.1–14.2 on average). For Canton-S, the peak was delayed during SI (8.9) with *ε* = 8 mm (green arrow, Figure 6b) while it was observed earlier during P-S with *ε* = 4 mm (green arrow, Figure 6a). For both threshold distances, decrease in cluster number was observed during SII (Orange arrows, Figure 6a,b). The cluster number decreased during PI–PIII (7.8, 7.1, and 6.6, respectively) for Canton-S, whereas for *tab2^201Y^*, the cluster number linearly increased with *ε* = 8 mm during this period (Figure 6b). Overall differences in the cluster numbers were observed for *tab2^201Y^* compared with Canton-S. For *tab2^201Y^*, the cluster number was low during PI and continuously increased until SII for both threshold distances (Figure 6a,b). In addition, for *tab2^201Y^*, the SD range was generally broad during the scotoperiod with *ε* = 4 mm and during the photoperiod with *ε* = 8 mm.

**Figure 7:**
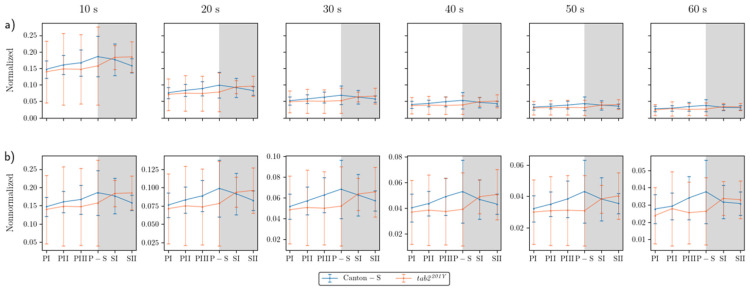
*I*-index of cumulated movement positions in different time window sizes for Canton-S and *tab2^201Y^ D. melanogaster*. (**a**) Normalized values across time window sizes and (**b**) closeup of the curves.

The overall dispersion patterns for the movement positions were examined using the *I*-index. Because the *I*-index globally measures the dispersion pattern focusing on individual isolation over the entire observation arena, determination of local spatial units was not necessary. Figure 7a presents the *I*-index for the cumulated movement positions across the entire observation arena for time windows of 10 s to 60 s under normalization. Generally, the values were narrow in a low range. The *I*-index was higher at a window size of 10 s (peaking at 0.19), decreasing dramatically as the time window increased from 20 s (peaking at 0.10) (Figure 7a).

The trend in the *I*-index values over time was generally consistent between the time windows compared with the non-normalized data (Figure 7b). The *I*-index peaked during P-S (0.04–0.19) for Canton-S, indicating that isolation of individuals was lowest during P-S. For *tab2^201Y^*, the *I*-index was low during the photoperiod (0.03–0.15) and then increased during the scotoperiod (0.03–0.18). The *I*-index across the light phase followed a similar pattern to that for speed, for both strains (Figure 4a). For Canton-S, the *I*-index and the speed both peaked during the same light phase (P-S), and the trend in *I*-index values over time was also similar to that of the cluster numbers, with ε = 4 mm (Figure 6a). The pattern of change in the *I*-index for *tab2^201Y^* was similar to that in speed in the overall observation arena (Figure 4a). The SD range for the *I*-index was broader overall for *tab2^201Y^* than for Canton-S and during the photoperiod than during the scotoperiod.

**Figure A2:**
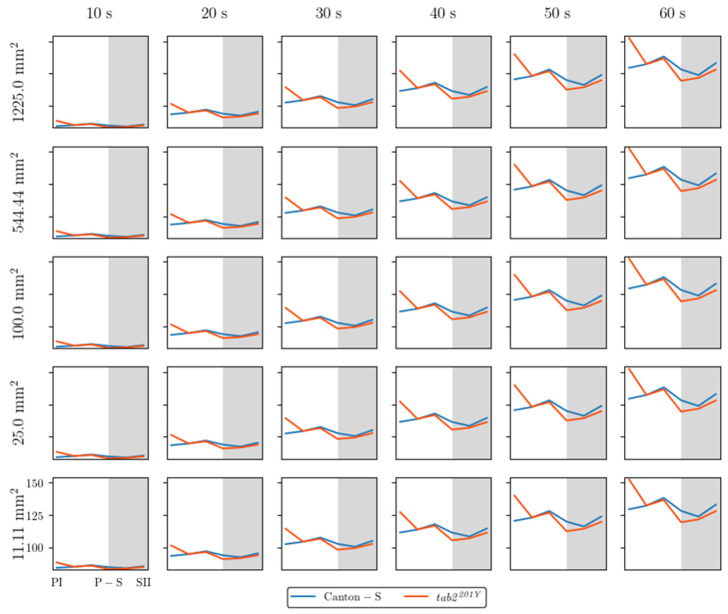
MC of cumulated movement positions across different levels of lattice size and time window sizes with normalization for Canton-S and *tab2^201Y^ D. melanogaster*.

While the *I*-index describes the degree of individual isolation, MC represents the local crowdedness within a particular spatial unit. MC was obtained according to time window sizes between 10 s and 60 s and spatial scales between 11.1 mm^2^ and 1225.0 mm^2^ to determine the optimal unit size for space and time (Figure A2). Overall, the trend over time for MC was similar between the spatial size and time windows. However, the MC values gradually increased with the time window size (Figure A2) in a manner similar to the cluster numbers (Figure A1).

The trends in the MC values across the light phases were selected for detailed comparison, with a spatial unit size of 25 mm^2^ and 100 mm^2^ and a window size of 30 s (Figure 6c,d). The overall trends over time were similar between the two spatial unit sizes and the two strains. These trends over time for the MC were also similar to that for the duration rate overall in the food-provision and center-diffusion areas (Figure 5a,b), with two peaks observed during the photoperiod and scotoperiod (blue and orange arrows, respectively, Figure 6c,d).

A slight difference was observed between Canton-S and *tab2^201Y^* during PI with a spatial unit size of 100 mm^2^, being higher for *tab2^201Y^* than for Canton-S during this phase (Figure 6d). The SD range was also broader overall for *tab2^201Y^* than for Canton-S, especially during the photoperiod.

**Figure A3:**
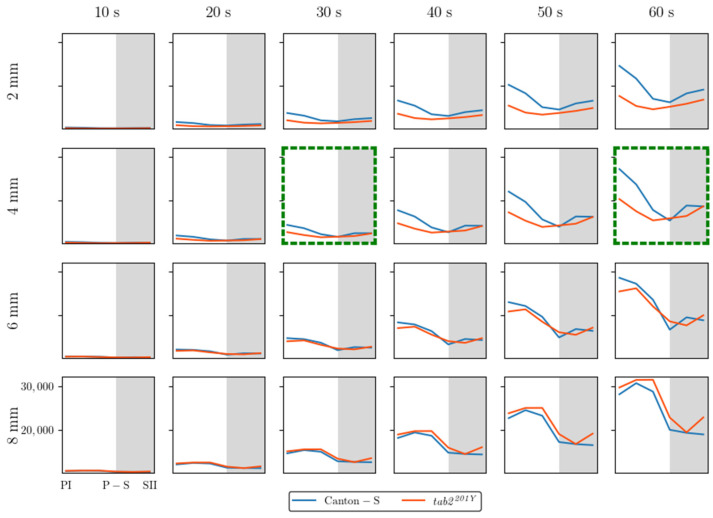
SSI of cumulated movement positions across different levels of time window size with normalization for Canton-S and *tab2^201Y^ D. melanogaster*.

The SSI was also measured across different threshold distances and time window sizes, as listed in Figure A3. While the SSI exhibited generally similar trends, the values increased as both the spatial distance and the time window size increased. With a threshold distance of 6 mm or lower, the SSI was higher for Canton-S and lower for *tab2^201Y^*. However, with a threshold distance of 8 mm, the SSI was lower for Canton-S and higher for *tab2^201Y^* (see the two green dotted rectangles shown as examples in Figure A3).

Although 4 mm is close to the critical distance for the SSI for *Drosophila* [2,39], for the purpose of comparison, we also investigated the SSI with a threshold distance of 8 mm with the same time unit of 30 s (Figure 6e,f). The shape of the trend in the SSI with a threshold distance of 4 mm was opposite to that for the cluster number (Figure 6a) and similar to that for the MC (Figure 6c). The minimum SSI was observed during P-S (purple arrow, Figure 6e). Genetic differences in the SSI were found at the threshold distance of 4 mm; in particular, the SSI values were substantially lower for *tab2^201Y^* than for Canton-S, especially during the photoperiod (Figure 6e).

The trend in the SSI across the light phases at a threshold distance of 8 mm exhibited different patterns, with high values during PII~PIII (Figure 6f). The SSI values were remarkably similar between the two strains, with a maximum during PII (10,637.24) and a minimum (5082.12) during SII for Canton-S, compared with a maximum during PIII (10,997.26) and a minimum during SI (5116.39) for *tab2^201Y^*. SDs were higher overall for *tab2^201Y^* than for Canton-S, with SDs for this strain exceptionally high in the photoperiod at a threshold distance of 8 mm.

### 3.4. Comparison of the Parameters Between Micro-Areas

**Figure 8:**
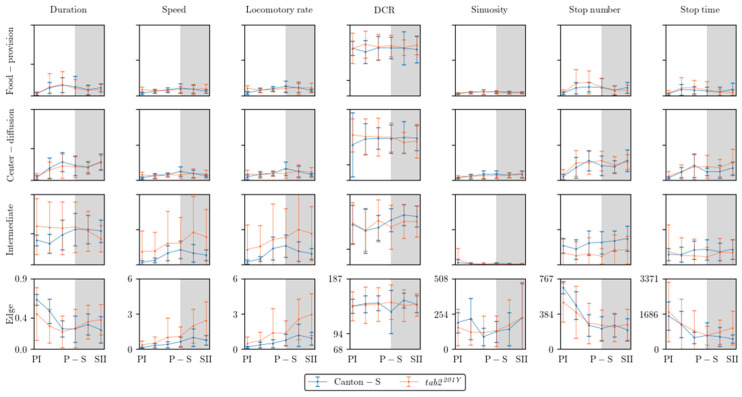
Movement parameters in different micro-areas in the observation arena across light phases for Canton-S and *tab2^201Y^ D. melanogaster* (normalized across micro-areas).

Figure 8 presents an overall view of the movement parameters with normalization for the micro-areas, with a time window size of 30 s. The trends in the movement parameters over time were generally similar between the food-provision and center-diffusion areas, while these trends were variable in the intermediate and edge areas. In particular, the motility and sessility parameters were more variable between the light phases in the intermediate and edge areas (Figure 8). Sinuosity exhibited high variability in the edge area, while it was more stable with low values in the other micro-areas. The DCR was consistently observed within a limited range around an average of 140.9°/s (though the change in direction, i.e., right or left, was not considered) across the light phases, indicating that large directional changes were observed during the 1 s time unit for group movement. Genetic differences in the motility parameters were also clearly observed in the intermediate and edge areas.

**Figure 9:**
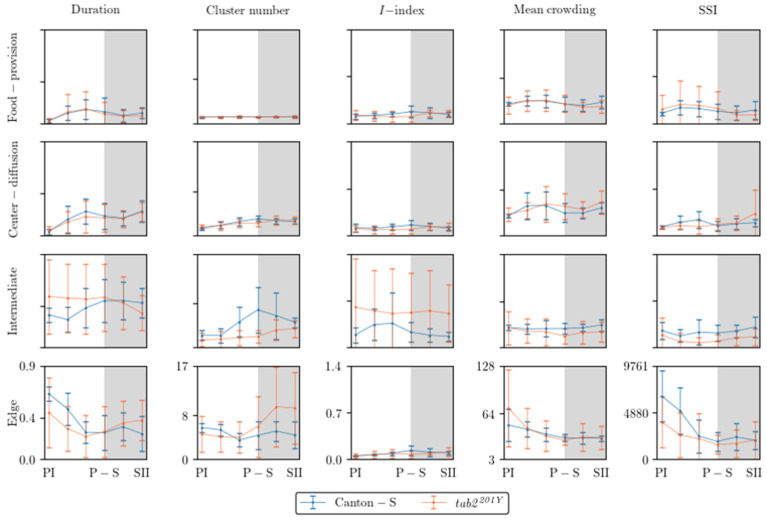
Dispersion parameters in different micro-areas in the observation arena across light phases for Canton-S and *tab2^201Y^ D. melanogaster* (normalized across micro-areas).

Figure 9 shows an overview of the dispersion parameters with normalization for the micro-areas with a time window size of 30 s and a threshold distance of 4 mm for cluster numbers and SSI, a lattice size of 25 mm^2^ for MC, and the entire observation arena (19,600.0 mm^2^) for the *I*-index. Similar to the movement parameters, the dispersion parameters were more variable in the intermediate and edge areas. The cluster number and the *I*-index were high in the intermediate area for Canton-S in accordance with the duration rate, while the cluster numbers were low in the food-provision and center-diffusion areas. The *I*-index was particularly high in the intermediate area, especially for *tab2^201Y^*, indicating a low degree of individual isolation. The cluster numbers and SSI were characterized by high values with a difference between the two strains at the edge of the area (Figure 9).

**Figure 10:**
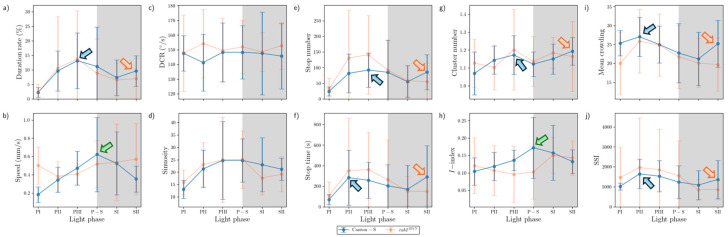
Movement and dispersion parameters in the food-provision area across light phases for Canton-S and *tab2^201Y^ D. melanogaster*. (**a**) Duration rate, (**b**) speed, (**c**) DCR, (**d**) sinuosity, (**e**) stop number, (**f**) stop time, (**g**) cluster number, (**h**) *I*-index, (**i**) MC, and (**j**) SSI.

**Figure 11:**
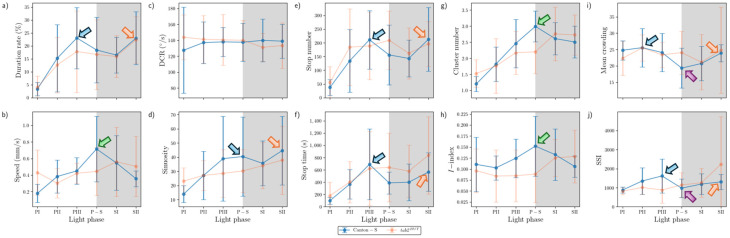
Movement and dispersion parameters in the center-diffusion area across light phases for Canton-S and *tab2^201Y^ D. melanogaster*. (**a**) Duration rate, (**b**) speed, (**c**) DCR, (**d**) sinuosity, (**e**) stop number, (**f**) stop time, (**g**) cluster number, (**h**) *I*-index, (**i**) MC, and (**j**) SSI.

**Figure 12:**
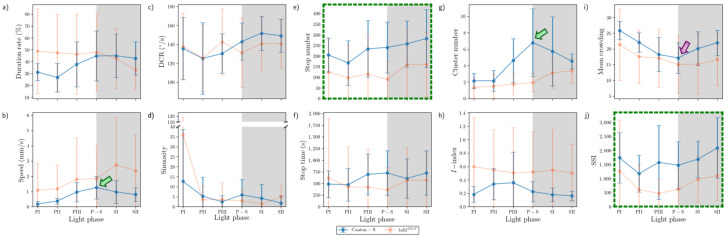
Movement and dispersion parameters in the intermediate area across light phases for Canton-S and *tab2^201Y^ D. melanogaster*. (**a**) Duration rate, (**b**) speed, (**c**) DCR, (**d**) sinuosity, (**e**) stop number, (**f**) stop time, (**g**) cluster number, (**h**) *I*-index, (**i**) MC, and (**j**) SSI.

**Figure 13:**
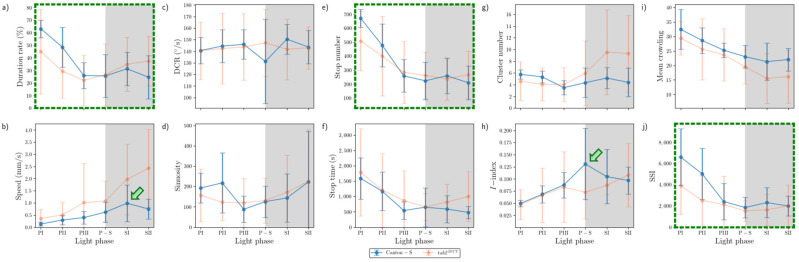
Movement and dispersion parameters in the edge area across light phases for Canton-S and *tab2^201Y^ D. melanogaster*. (**a**) Duration rate, (**b**) speed, (**c**) DCR, (**d**) sinuosity, (**e**) stop number, (**f**) stop time, (**g**) cluster number, (**h**) *I*-index, (**i**) MC, and (**j**) SSI.

The movement and dispersion parameters are presented together for each micro-area in Figure 10, Figure 11, Figure 12 and Figure 13, focusing on trends over time. The trends for the parameters were similar overall between the resource supply areas (i.e., food and moisture), while those in areas related to activity (i.e., open space in the intermediate area and edge area) varied greatly.

Temporally co-occurring trends were found for some measured parameters for Canton-S across the light phases depending on the micro-area. In the food-provision area, two major trends were observed over time. The first trend was a single peak for speed (0.6 mm/s) and the *I*-index (0.2) during P-S (green arrows, Figure 10b,h), while the second trend was two peaks during PII~PIII and SII for the duration rate (PIII: 13.1%; SII: 9.6%), stop number (PIII: 135.9; SII: 55.5), stop time (PII: 357.0 s; SII: 145.0 s), cluster number (PIII: 1.2; SII: 1.2), MC (PII: 27.0; SII: 25.2), and SSI (PII: 1645.5; SII: 1367.5) (blue and orange arrows, Figure 10a,e–g,i,j). Most of the dispersion parameters except for the *I*-index exhibited two peaks in the food-provision area. The temporally co-occurring parameters with two peaks reflected local aggregations for feeding along with maximum durations in the food provision area.

Sinuosity exhibited a unique pattern with an early increase during the photoperiod to reach the highest level during PIII~P-S, followed by a slight decrease during the scotoperiod for Canton-S in the range of 13.0–24.7 (Figure 10d). The DCR was stable across the light phases at around 148.6°/s (Figure 10c).

Comparing Canton-S and *tab2^201Y^*, the trends according to the light phase were substantially different for the speed and *I*-index, whereas similar patterns were observed over time for the other parameters. While Canton-S had a maximum speed during P-S (0.6 mm/s) and a clear diel difference, the diel difference for *tab2^201Y^* was less distinct, with low values during the photoperiod (0.4 mm/s) and a high value during the scotoperiod (0.6 mm/s) (Figure 10b). The *I*-index was low overall (0.10–0.12) until P-S, followed by an increase during SI~SII (0.14–0.15) for *tab2^201Y^*, which was in contrast to the single peak during P-S for Canton-S (Figure 10h). The stop number and stop time also differed, with higher averages during the photoperiod (135.9 and 357.0 s, respectively) and lower averages during SII (55.5 and 149.5 s, respectively) for *tab2^201Y^* than for Canton-S (Figure 10e,f).

SDs were variably expressed according to parameters, light phases, and micro-areas (vertical bars, Figure 10, Figure 11, Figure 12 and Figure 13). Since the parameter values were not normalized, the degree of variability cannot be objectively compared with other parameters in these figures. The quantitative degree of variability, expressed as coefficient of variation (CV; ratio of SD to mean) of measured parameters, is discussed in the Section 3.5, *Data Variability and Statistical Differentiation*. In Figure 10, Figure 11, Figure 12 and Figure 13, relative SD sizes across light phases are described for different parameters in the two strains.

In the food-provision area, for *tab2^201Y^*, distinctively high SDs were observed in the photoperiod for duration rate, stop number and time, and SSI (Figure 10a,e,f,j) and in the scotoperiod for speed (Figure 10b). For *tab2^201Y^*, the cluster numbers had intermittently high SDs in the photo- and scotoperiods (Figure 10g). Outstanding SDs were relatively fewer for Canton-S than for *tab2^201Y^*. For Canton-S, SDs for speed, MC, and *I*-index were high in the scotoperiod (Figure 10b,j,i) and SDs for duration rate and sinuosity were intermittently high in the photoperiod (Figure 10a,d).

The parameter trends in the center-diffusion area (Figure 11) were generally similar to those for the food-provision area (Figure 10). Single peaks during P-S were observed for the speed (0.7 mm/s) and *I*-index (0.15) for Canton-S (green arrows, Figure 11b,h). A single peak was also observed for the cluster number (2.99) during P-S in the center-diffusion area (green arrow, Figure 11g), whereas the cluster number had double peaks in the food-provision area (Figure 10g). Sinuosity also exhibited double peaks in the center-diffusion area during P-S (40.5) and SII (44.7) (blue and orange arrows, Figure 11d), contrary to the trend of sinuosity in the food-provision area that showed the highest level during PIII~P-S for Canton-S (Figure 10d). Similar to the case of the food provision area, the parameters with two peaks reflected local aggregations for obtaining humidity in accordance with maximum durations in the center-diffusion area (blue and orange arrows, Figure 11a). Notably, the minimum for MC and SSI was observed during P-S (purple arrows, Figure 11i,j), indicating minimum local aggregation, while speed was at its maximum (green arrow, Figure 11b).

The sinuosity, cluster number, MC, and SSI (Figure 11d,g,i,j) were considerably different for *tab2^201Y^* compared with Canton-S in the center-diffusion area, while the other parameters had similar trends between two strains to those observed in the food-provision area. The sinuosity exhibited two peaks over time for Canton-S and a linear increase toward the scotoperiod for *tab2^201Y^* (Figure 11d). MC and SSI also had two peaks for Canton-S, whereas these peaks were not observed for *tab2^201Y^* (Figure 11i,j). The cluster number had a single peak during P-S for Canton-S, which was in contrast to the linear increase observed for *tab2^201Y^* (Figure 11g). Although similar, minor differences were found in the stop number and stop time between the two strains, with higher stop numbers (PII~P-S: 167.2; SII: 213.2 on average) and stop times (P-S: 641.1 s; SII: 839.4 s on average) for *tab2^201Y^* (Figure 11e,f).

SDs in the center-diffusion area were calculated, with similarities and differences compared with the food-provision area. For *tab2^201Y^*, high SDs for duration rate and stop number were observed in the photoperiod (Figure 11a,e) and for speed, MC, and SSI in the scotoperiod (Figure 11b,i,j). The stop time was intermittently high in both the photo- and scotoperiods (Figure 11f). For Canton-S, high SDs were observed for duration rate, DCR, and stop time in the photoperiod (Figure 11a,c,f) and for speed in the scotoperiod (Figure 11b). For Canton-S, the stop numbers were intermittently high in both the photo- and scotoperiods (Figure 11e). It was noted that high SDs for duration rate and speed occurred in the photoperiod and scotoperiod, respectively, in both strains, in the food-provision and center-diffusion areas (Figure 10a,b and Figure 11a,b). Extremely high SDs were observed for MC and SSI during SII for *tab2^201Y^* compared with Canton-S (Figure 11i,j), whereas the SD of DCR was outstandingly high during PI for Canton-S compared with *tab2^201Y^* (Figure 11c).

In the intermediate area, substantial differences were found in the parameter trends across the light phases (Figure 12) compared with the areas for resource provision. For Canton-S, increases were observed for the duration rate (21.5% on average), speed (0.3 mm/s on average), stop number (81.9 on average), and stop time (199.2 s on average), compared with the center-diffusion area, while a decrease was observed for sinuosity (28.1 on average). The trend for speed in the intermediate area (green arrow, Figure 12b) was similar to that observed for the food-provision and center-diffusion areas, with a single peak during P-S for Canton-S. The two peaks observed for many parameters during the photoperiod and scotoperiod in the food-provision and center-diffusion areas were not observed in the intermediate area.

In the intermediate area, a number of parameters were low during the photoperiod and high during the scotoperiod, including the duration rate (photoperiod: 31.9%; scotoperiod: 43.9%), speed (0.5 mm/s and 0.9 mm/s, respectively), and stop number (202.0 and 269.9, respectively) for Canton-S (Figure 12a,b,e). Except for the high value during PI (12.7), sinuosity was generally stable (4.0 on average) afterward (Figure 12d). The DCR was slightly variable (139.1 on average) in the intermediate area (Figure 12c).

Dispersion parameter patterns were also substantially different from those of the food-provision and center-diffusion areas (Figure 12g–j). The cluster number had a single peak during P-S (6.8), matching the peak for speed (Figure 12b,g). The trends for the *I*-index, MC, and SSI were different overall from each other for Canton-S. The *I*-index had a peak early during PIII (Figure 12h). For MC, the minimum (17.1) was observed in the intermediate area (purple arrow, Figure 12i), like the case of center-diffusion area (Figure 11i). Temporally co-occurring trends were also observed between parameters; the trend in the SSI was very similar to that in stop numbers (dotted green rectangles, Figure 12e,j).

Differences in the parameters between Canton-S and *tab2^201Y^* were observed in the intermediate area. The duration rate differed between light phases, being higher in the photoperiod (47.5% on average) and lower in the scotoperiod (37.9% on average) for *tab2^201Y^* than for Canton-S (Figure 12a). Although the trend in the speed was similar between the two strains in the center-diffusion area (Figure 11b), the speed of *tab2^201Y^* (1.8 mm/s) was substantially higher overall than for Canton-S (0.8 mm/s) (Figure 12b) in the intermediate area. This suggested the high speeds observed for *tab2^201Y^* overall originated from high speeds in the intermediate area, especially during the scotoperiod. In accordance with this, the number of stops was consistently lower for *tab2^201Y^* (124.3) than for Canton-S (231.1) across the light phases (Figure 12e). The stop time, however, did not differ significantly between the two strains except for a minor increase during PIII (697.1 s) and P-S (723.4 s) for Canton-S compared with *tab2^201Y^* (419.4 s and 362.7 s, respectively) (Figure 12f).

For *tab2^201Y^*, comparing to the center-diffusion area, increases in parameter values in the intermediate area were observed for duration rate (44.4%) and speed (1.8 mm/s) (Figure 12a,b), while decreases were observed for sinuosity (8.8), stop numbers (124.3), and stop time (494.3 s) on average (Figure 12d–f). Sinuosity was exceptionally higher during PI for *tab2^201Y^* (36.1) than for Canton-S (12.7), indirectly indicating a high degree of searching around activity for *tab2^201Y^*. The patterns for stop numbers and SSI over time were remarkably similar for both *tab2^201Y^* and Canton-S (dotted green rectangles, Figure 12e,j), suggesting that the balance between attraction and repulsion with regard to neighboring individuals was preserved through frequent stops in both the wild type and the mutant.

The SD patterns in the intermediate area were substantially different from those observed in the food-provision and center-diffusion areas. For Canton-S, SDs of many parameters were relatively stable, including duration rate, speed, stop numbers and time, and MC (Figure 12a,b,e,f,i). SDs were intermittently high in the photoperiod for DCR, sinuosity, and *I*-index (Figure 12c,d,h), and high in the scotoperiod for cluster numbers (Figure 12g). The SD of SSI was sporadically high in both the photo- and scotoperiods for the same strain (Figure 12j). For *tab2^201Y^*, SDs were high overall for duration rate, speed, stop time, *I*-index, and MC (Figure 12a,b,f,h,i), with lower cluster numbers (Figure 12g) compared with Canton-S. For *tab2^201Y^*, SDs for duration rate, DCR, and stop time were high in the photoperiod at variable times(Figure 12a,c,f), and the SD of their speed was intermittently high in both the photo- and scotoperiods (Figure 12a). It was noted that SDs for sinuosity were exceptionally high during PI for both strains (Figure 12d).

The parameters in the edge area were substantially different from those in the other micro-areas (Figure 13). Peaks often observed in the resource provision areas were not found in the edge area for Canton-S. The peak for the speed (1.0 mm/s) was observed slightly later during SI in the edge area (green arrow, Figure 13b), compared with the peak during P-S in the other areas.

A substantial increase on average sinuosity (165.2 on average) was observed in the edge area compared with the intermediate area (5.4 on average) for Canton-S (Figure 12d and Figure 13d). A clear increase was also observed for the average SSI (3371.9 on average) in the edge area (Figure 13j) compared with the intermediate (1624.2 on average) and other areas, indicating a strong aggregation in the area close to the boundary in the observation arena. Exceptionally high values were observed for the average duration rate (55.9% on average), stop numbers (573.2 on average), and stop time (1377.9 s on average) during PI~PII in the edge area (Figure 13a,e,f). These values later stabilized at an average of 26.9% for the duration rate, 237.9 for stop numbers, and 566.7 s for the stop time in the edge area. The DCR was stable at 142.9°/s on average, although slight variation was observed during P-S.

The dispersion parameters in the edge area were also substantially different from those in the other micro-areas (Figure 13g–j). A peak during P-S was observed for the *I*-index (0.13) for Canton-S (green arrow, Figure 13h), matching the peak for speed (green arrow, Figure 13b). While the cluster number (4.7 on average) was stable across the light phases, high values were observed in early photoperiod for MC (48.5) and the SSI (6610.2) for Canton-S, but these decreased and stabilized during the scotoperiod (31.3 and 2158.5, respectively, on average) (Figure 13i,j).

Differences in the behaviors of the two strains were also observed in the edge area. Regarding the movement parameters, the average duration rate was lower during the photoperiod (32.2% on average) and higher during the scotoperiod (36.2% on average) for *tab2^201Y^* compared with Canton-S (45.9% and 28.0% respectively, on average) (Figure 12a). The speed was also substantially higher across the light phases for *tab2^201Y^* than for Canton-S (Figure 12b). This difference was not great during the PI, but it continuously increased until SII, reaching 2.4 mm/s for *tab2^201Y^* compared with 0.8 mm/s for Canton-S (Figure 13b). Together with the faster speeds in the intermediate area, the speed in the edge area during the scotoperiod contributed greatly to the increase in the total speed of *tab2^201Y^* (Figure 4a). The trend in stop numbers over time was similar between the two strains in the edge area (Figure 13e), whereas the stop numbers differed between the two strains especially in the photoperiod in the intermediate area (Figure 12e). However, the DCR, sinuosity, stop numbers, and stop time were similar overall between the two strains (Figure 13c,e,f).

Temporally co-occurring patterns were also observed between parameters in the edge area. The patterns over time for the duration rate, stop number, and SSI were very similar between Canton-S and *tab2^201Y^* (green dotted rectangles, Figure 13a,e,j). The stop number and SSI were in accordance in the intermediate area, as stated above (green dotted rectangles, Figure 12e,j), while the duration rate was added to this group in the edge area, with very high values observed during the early photoperiod. This temporal co-occurrence trend for stop numbers and SSI in the areas related to activity persisted between strains.

The data variability patterns in the edge area were broadly similar to those in the intermediate area while allowing for some local differences. The SDs for *tab2^201Y^* were high for duration rate, speed, DCR, stop time, cluster numbers, *I*-index, and MC, compared with Canton-S (Figure 13a–c,f–i), similar to the case of the intermediate area. For *tab2^201Y^*, the SDs increased correspondingly with the values of speed and cluster increasing as the time progressed toward the scotoperiod (Figure 13b,g). For Canton-S, the SDs of many parameters were relatively stable, including duration rate, speed, DCR, stop numbers and time, cluster numbers, and MC (Figure 13a–c,e–g,i), broadly similar to the case of intermediate area. For Canton-S, SDs were intermittently high in the photoperiod for SSI (Figure 13j) and high in the scotoperiod for *I*-index (Figure 13h), and the SD of sinuosity was sporadically high in both the photo- and scotoperiods in the same strain (Figure 13d).

In summary, the following common patterns were observed for the movement parameters across the light phases for Canton-S (Figure 10, Figure 11, Figure 12 and Figure 13):

1. A single peak during P-S for speed in most micro-areas except the edge area (green arrows, Figure 10, Figure 11 and Figure 12);
2. Two peaks during the mid-photoperiod and end of the scotoperiod for the duration rate, stop numbers, and stop time in the food-provision and center-diffusion areas (blue and orange arrows, Figure 10 and Figure 11);
3. A peak during the early photoperiod followed by a minimum during the scotoperiod for the duration rate, stop numbers, and stop time in the edge area (Figure 13a,e,f);
4. Low values during the photoperiod and high values during the scotoperiod for the duration rate, DCR, and stop time in the intermediate area (Figure 12a,c,e) and for speed in the edge area (Figure 13b);
5. A peak during the early photoperiod along with a minimum during the scotoperiod for the duration rate, stop numbers, and stop time in the edge area (Figure 13a,e,f).

Dispersion parameters also had frequently observed patterns across the light phases for Canton-S, as follows:

1. A single peak during P-S for the *I*-index in the food-provision, center-diffusion, and edge areas (green arrows, Figure 10h, Figure 11h and Figure 13h) and for the cluster number in the center-diffusion and intermediate areas (green arrows, Figure 11g and Figure 12g);
2. Two peaks during the mid-photoperiod and the end of the scotoperiod for the cluster numbers, MC, and SSI in the food-provision area (blue and orange arrows, Figure 10g,i,j) and for MC and SSI in the center-diffusion area (blue and orange arrows, Figure 11i,j);
3. A peak during the early photoperiod followed by a minimum during the scotoperiod for MC and the SSI in the edge area (Figure 13i,j);
4. A minimum during P-S for MC and the SSI in the center-diffusion area (purple arrows, Figure 11i,j) and for MC in the intermediate area (purple arrow, Figure 12i).

### 3.5. Data Variability and Statistical Differentiation

**Figure 14:**
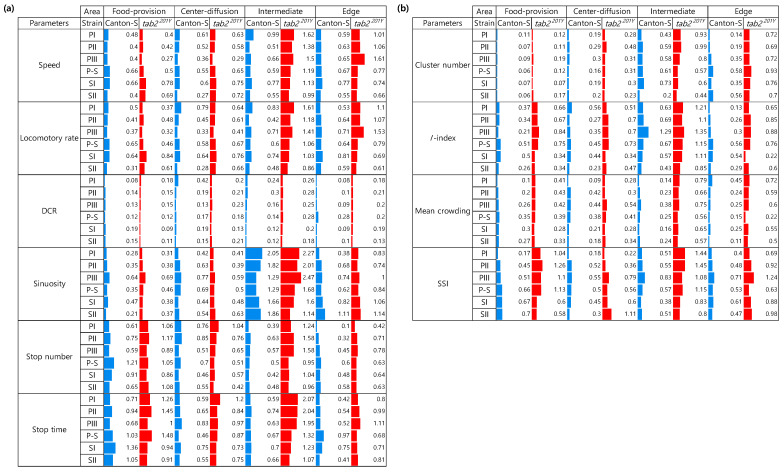
CV for group movement parameters in each micro-area across light phases for Canton-S and *tab2^201Y^ D. melanogaster*. (**a**) Movement and (**b**) dispersion parameters (Blue: Canton-S; Red: *tab2^201Y^*).

Because high variability was observed for the movement and dispersion parameters, the CV was used to compare the degree of variation in these parameters according to the micro-area, light phase, and strain. For the movement parameters, sinuosity overall exhibited high CVs for both strains (0.21–2.05 for Canton-S and 0.31–2.47 for *tab2^201Y^*) (Figure 14a). In contrast, CVs were low overall for the DCR for both strains (0.08–0.42 and 0.09–0.28, respectively).

For Canton-S, CVs were high overall in the areas related to activity compared with the areas related to resource supply. In the food-provision area, the stop numbers (0.61–1.21) and stop time (0.68–1.36) had higher ranges than the other parameters (Figure 14a). Stop numbers (0.46–0.85) and stop time (0.59–0.83) showed slightly high range of CVs than speed (0.27–0.61) and locomotory rate (0.28–0.79) in the center-diffusion area.

For Canton-S, in the intermediate area, sinuosity (1.29–2.05) had the highest CV followed by speed (0.51–0.99) and locomotory rate (0.42–0.83) (Figure 14a). In the edge area, the CV range was relatively low, being highest for sinuosity (0.38–1.11) and the locomotory rate (0.53–0.81).

The CVs for the movement parameters for *tab2^201Y^* were higher overall than those for Canton-S, while the CV trends within the micro-areas were similar (Figure 14a). Higher CVs were found in the intermediate area than in the other micro-areas, with many parameters exhibiting CVs over 1.0, including sinuosity (1.14–2.47), stop time (1.07–2.07), speed (0.99–1.62), locomotory rate (0.86–1.61), and stop number, (0.95–1.58 for *tab2^201Y^* (Figure 14). Differences between the two strains were also observed in the edge area. While the CVs for sinuosity were not much different, as stated above, those for the speed (0.66–1.61) and locomotory rate (0.61–1.53) were higher for *tab2^201Y^* than for Canton-S (0.55–0.77 and 0.53–0.81, respectively).

Figure 14b presents the CVs for dispersion parameters according to the micro-area and light phase. The CVs for the dispersion parameters were generally lower than those for the movement parameters. Among the micro-areas, the CVs were higher in the intermediate area for the SSI (0.38–0.83 for Canton-S and 0.80–1.45 for *tab2^201Y^*) and the *I*-index (0.43–1.29 and 0.85–1.35, respectively) compared with the other indices. The cluster number had low CVs in the food-provision (0.06–0.11 for Canton-S and 0.07–0.19 for *tab2^201Y^*) and center-diffusion areas (0.16–0.30 and 0.23–0.48, respectively) compared with the other micro-areas (Figure 14b).

Due to the high variability of the parameters, statistically significant differences were observed in cases where the values of the parameters varied strongly. In addition to this data variability, the observed behavior in this study had the structural property of measurement dependence between two factors coupled in the spatio-temporal domain (see Section 2.4, *Statistical Analysis*). To accommodate the coupled dependence, we conducted two-way repeated measurement ANOVA. Table 1 shows statistical differences in parameters in coupled dependence within and between trials for micro-areas and light phases.

Within these trials, for Canton-S, most movement parameters were highly significant (*p* ≤ 0.010) with the non-significant exceptions including DCR (*p* = 0.349), MC (*p* ≤ 0.081), and *I*-index (*p* ≤ 0.073) for micro-areas (Table 1a). For light phases, probabilities for alpha errors were higher overall than for micro-areas. Only the stop numbers (*p* = 0.002) had high significance, followed by significance for SSI (*p* = 0.040), speed (*p* = 0.044), and locomotory rate (*p* = 0.049), while other parameters were not significant. Cofactors between micro-areas and light phases had high significance, including stop numbers (*p* = 0.000), stop time (*p* = 0.004), and SSI (*p* = 0.004), followed by significance at a low level for cluster numbers (*p* = 0.025). The other parameters were not significant for Canton-S (Table 1a). Between the trials, all parameters were highly significant (0.000 ≤ *p* ≤ 0.001) for both strains, indicating strong differences between the trials (Table 1b).

For *tab2^201Y^*, less significance was observed than for Canton-S. For micro-areas, high significance was found for stop numbers (*p* ≤ 0.000) and sinuosity (*p* ≤ 0.009), while MC (*p* = 0.017), stop time (*p* = 0.027), cluster numbers (*p* = 0.047), and SSI (*p* = 0.043) had significance (Table 1a). For light phases, all parameters were not significant (0.127 ≤ *p* ≤ 0.904). Cofactors between micro-areas and light phases were not significant for all parameters. Between trials, movement parameters were highly significant, while MC, locomotory rate and speed (0. 025 ≤ *p* ≤ 0.039) showed less significance. Overall the degree of significance was lower than for Canton-S (Table 1b).

To investigate how group behaviors were differentiated by micro-area and light phase, the Friedman test was conducted (see Section 2.4, *Statistical Analysis*). For Canton-S, a majority of parameters were highly significant (*p* ≤ 0.01) overall during the early photoperiod (PI and PII) and scotoperiod (SI and SII) and less strong during the late photoperiod (PIII) and the change from the photoperiod to the scotoperiod (P-S) (Table 2a). Motility parameters had no significance at PI and PII, while sessility parameters showed no significance during SII (Table 2a). For *tab2^201Y^* the general trend of significance was like that of Canton-S, with slight variation (Table 2b). The beginning of the photoperiod (PI) and end of the scotoperiod (SII) had several parameters of high significance. In both strains, motility parameters had no significance during PI~PII while SSI had no significance during PIII and P-S (Table 2a).

Subsequently, statistical differences with dependent measurements of light phases were calculated in separate data sets for the micro-areas according to the Friedman test, as shown in Table 3 (See Section 2.4 *Statistical analysis*). For Canton-S, the center diffusion area had probabilities of high significance (*p* ≤ 0.008) in all motility and sessility parameters except DCR (*p* = 0.744) (Table 3a). The edge area also had many parameters with high significance, while the intermediate area had significance in speed and locomotory rate (*p* ≤ 0.001). For *tab2^201Y^*, the trends of significance were somewhat different, slightly less significant than for Canton-S especially in the center-diffusion area. Only one parameter, cluster numbers (*p* = 0.002) had high significance, followed by significance for stop time (*p* = 0.024) and stop numbers (*p* = 0.037) in the center-diffusion area (Table 3b), whereas large number of parameters were significant for Canton-S (Table 3a). In the food-provision and intermediate areas, parameters were also not significant except *I*-index (*p* = 0.023). In the intermediate area, speed (*p* = 0.004) and locomotory rate (*p* = 0.004) were highly significant while cluster number (*p* = 0.035) and MC (*p* = 0.047) were significant. Similarly, in the edge area, speed, locomotory rate, MC, and *I*-index (0.001 ≤ *p* ≤ 0.010) were highly significant and stop numbers and cluster number were significant (*p* = 0.042~0.045).

After confirming statistical significance in the Friedman test, multiple comparison tests were conducted via the Wilcoxon signed-rank test [60], applicable to rank, and paired permutation test [61], applicable to the mean (see Section 2.4, *Statistical Analysis*). To secure significance in a conservative aspect, probabilities of alpha error were divided by the number of paired tests for comparison (6 for micro-areas and 15 for light phases). Final probabilities for determining alpha errors for significance were 0.0500/6 = 0.0083 for micro-areas and 0.05/15 = 0.00333 for light phases. The probabilities for statistics obtained from observation data ranged 0.008~1.000 for both micro-areas and light phases except for very limited cases. The criteria for the probabilities of alpha errors were lower than for the obtained probabilities. The parameters in almost all combinations of micro-areas and light phases were not significantly different overall, although significances in the total treatments were observed according to the Friedman test (Table 2 and Table 3).

**Figure 15:**
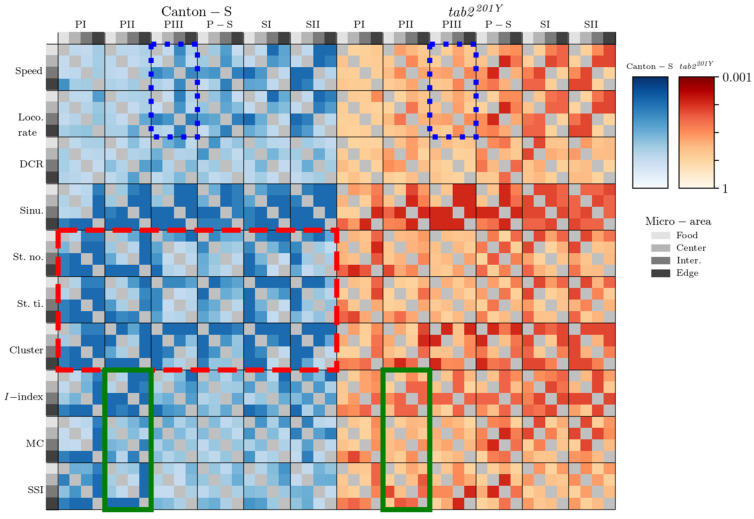
Statistical differentiation of movement and dispersion parameters across micro-areas for Canton-S and *tab2^201Y^ D. melanogaster* according to combined results from the Wilcoxon signed-rank and paired permutation tests. St. no.; Stop number: St. ti.; Stop time: Inter.; Intermediate.

**Figure 16:**
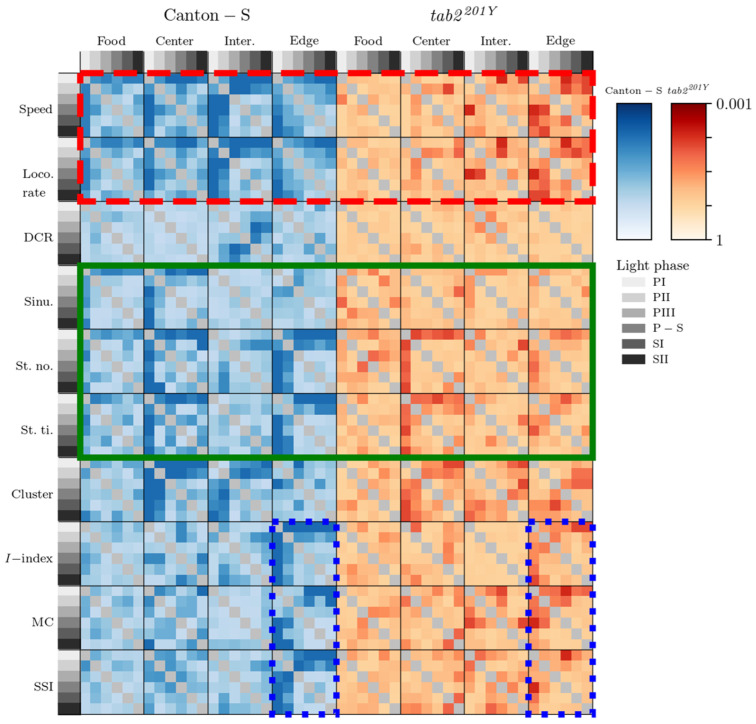
Statistical differentiation of movement and dispersion parameters across light phases for Canton-S and *tab2^201Y^ D. melanogaster* according to combined results from the Wilcoxon signed-rank and paired permutation tests. St. no.; Stop number: St. ti.; Stop time: Inter.; Intermediate.

To visualize the trends of relative difference between micro-areas and light phases although the data were not significant, the probabilities according to the Wilcoxon signed-rank and paired permutation tests in log (common) scales were averaged and visualized according to micro-area (separately in light phases) and light phase (separately in micro-areas), respectively (Figure 15 and Figure 16). Regarding differences in the micro-areas, sinuosity was most clearly differentiated in movement parameters between the micro-areas during the light phases for both strains, whereas for Canton-S, the DCR was not very different between these areas. Sessility (i.e., the stop number and stop time) and motility (i.e., the speed and locomotory rate) parameters exhibited some differentiation between the micro-areas, light phases, and strains (Figure 15).

The movement parameters, stop number, and stop time and the cluster number in the dispersion parameters were differentiated in the intermediate and edge areas during PI~PII and in the food-provision area during PIII–SII, for Canton-S (red dashed rectangle, Figure 15), indicating that the parameters for sessility and the cluster number were sensitive to differences in group behaviors between micro-areas. Regarding the dispersion parameters, differentiation was also observed for the *I*-index, MC, and SSI, primarily in the edge area during PI (the last column of each heatmap plot for each parameter). In contrast, the motility parameters were not as distinct as the other parameters, except for differences observed in the speed and locomotory rate during PIII (in the intermediate area; third column of the heatmap plot) and SII (in the intermediate and edge areas; third and fourth columns of the heatmap plots).

The mutant *tab2^201Y^* exhibited similar differentiation to Canton-S, but the degree of this differentiation was overall slightly weaker for both movement and dispersion parameters (Figure 15), confirming the broader data variability of parameters observed for *tab2^201Y^* (e.g., Figure 10, Figure 11, Figure 12 and Figure 13). Unlike with the *I*-index, the MC and SSI during PII observed for Canton-S was not observed for *tab2^201Y^* (two green solid rectangles, Figure 15). Differences in the speed and locomotory rate observed for Canton-S were not observed for *tab2^201Y^* (two blue dotted rectangles, Figure 15), indicating that differences in group behavior were weaker in the mutant. A slight difference was observed with mobility parameters between the two strains during P-S, and *I*-index during SI and SII (Figure 15).

Figure 16 presents differentiation between light phases for the movement and dispersion parameters in each micro-area in two strains. Parameter differences were consistently observed in the edge area (the vertical column matching the edge area for Canton-S, Figure 16), primarily during PI (first column within each heatmap plot). In the food-provision and center-diffusion areas, the motility and sessility parameters were differentiated, while the cluster numbers in the dispersion parameters were different in the center-diffusion area. The DCR was not different except for a minor difference in the intermediate area. The *I*-index, MC, and SSI were not differentiable between the micro-areas except in the edge area.

Differentiation between light phases for *tab2^201Y^* was observed less overall compared with Canton-S (Figure 16). No obvious differences were presented for *tab2^201Y^* except in the center-diffusion area. The differences observed for the sinuosity, stop numbers, and stop time for Canton-S were not observed for *tab2^201Y^*, with differences only in the center-diffusion area for the stop numbers and stop time (green solid rectangle, Figure 16). Strong differentiation was observed for the speed and locomotory rate for Canton-S, but this differentiation was not observed in most areas except for slight differences in the edge area for *tab2^201Y^* (red dashed rectangle, Figure 16). Similarly, the differentiation observed for the *I*-index, MC, and SSI in the edge area for Canton-S was not observed for *tab2^201Y^* (two blue dotted rectangles, Figure 16). These results indicate that the differences in the genetic make-up of *tab2^201Y^* more severely affected behaviors related to the light phases than to the micro-areas. It should be noted that Figure 15 and Figure 16 do not indicate statistical significance but illustrate the relative differences in parameters between micro-areas and light phases, as stated above.

Table 4 summarizes the statistical differences in the movement and dispersion parameters between Canton-S and *tab2^201Y^*, according to the Wilcoxon sign-rank and paired permutation tests (see Section 2.4, *Statistical Analysis*). The statistical differences highlighted in blue (*p* < 0.05) and green (*p* < 0.10) show possibilities of separation between parameters with high variability in two strains. Regarding the parameters, statistical differences were often observed in locomotory rate, stop numbers, SSI, and cluster numbers according to different light phases. In particular, the intermediate area had high significance in most parameters across the light phases. Motility and dispersion parameters were more different in the edge area, while dispersion parameters and sinuosity were statistically separated in the center-diffusion area. In the food-provision aera, parameters were less differentiated except for motility parameters and *I*-index (Table 4). The locomotory rate was slightly more statistically differentiable than the speed in the intermediate and edge areas in a broad range of light phases. Overall, statistical differentiation between two strains was observed according to parameters, different micro-areas, and light phases, matching the differences in the parameters observed between the micro-areas overall (Figure 10, Figure 11, Figure 12 and Figure 13).

## 4. Discussion and Conclusions

In the present study, the collective group behaviors of *D. melanogaster* in different micro-areas were characterized according to their movement and dispersion parameters. The parameters were systematically selected to investigate instantaneous movement (motility, sessility, and curvature) and dispersion (cluster numbers, *I*-index, MC, and SSI). The results confirmed the natural tendency for local enhancement in *D. melanogaster* [1,2] at a relatively low density, over the entire observation period (24 h).

Ten individuals were observed in the observation arena (14 cm × 14 cm × 2.63 mm) in this study, with a density of 0.05 indi./cm^2^. Similar studies in two dimensions (with a narrow height) in either a rectangular or circular shape were mostly conducted with density in the range of 0.3~1.0 indi./cm^2^ [2,28,34,36,37], matching 43.2~144.0 individuals in the observation arena with a size of 14 cm × 14 cm. Other studies have been performed with the density ranging 0.02~0.75 indi./cm^2^, focusing on measuring social space and revealing mechanosensory interactions [2,6]. In the present study, we aimed to see how collective behavior would arise among low-density groups while movement behaviors were observed continuously for a long period (one day).

Since density was low in the observation arena, space was sufficiently broad; overall crowdedness over the whole area was not observed in this study. Local cluster formation between individuals was presented, confirming aggregation mainly due to chemosensory cues, as previously reported in numerous studies [2,6,28,35,36]. It was noted that cluster numbers varied according to experimental conditions, micro-areas, light phases, and strains (Figure 10g, Figure 11g, Figure 12g and Figure 13g). For Canton-S, cluster numbers were in the high range (3.49–5.76) on average in the edge area, while the cluster numbers in intermediate area were variable between 2.15 and 6.79 on average, with a peak during P-S. However, the cluster numbers were substantially lower in the food-provision area with 1.07–1.19 on average. In the center-diffusion area, cluster numbers were variable in the low range between 1.20–2.99 on average, with a peak during P-S. For *tab2^201Y^*, cluster numbers were in a similar range to Canton-S in the resource provision areas. In the micro-areas related to activity, however, cluster numbers were substantially different in the mutant. In the intermediate area, cluster numbers were exceedingly low at 1.33–3.36 on average, whereas cluster numbers were notably higher in the scotoperiod at 9.32–9.56 on average for *tab2^201Y^*. The results overall indicated the existence of differences of local group formation according to different micro-areas and light phases in the two strains. In the present study, the number of individuals per cluster and cluster areas were not further investigated, since this research focused on comparing multiple parameters as an initial step indicating group behaviors in different micro-areas. Future studies are warranted regarding how clusters originate and change dynamically as time progresses in diverse experimental conditions in the spatio-temporal domain.

Interesting behavioral patterns were observed for Canton-S in the areas providing resources. In particular, a double-peak pattern consisting of a single peak during P-S (green arrows, Figure 4a,b; Figure 10e,h and Figure 11e,g,h) and two peaks during the mid-photoperiod and the end of scotoperiod (blue and orange arrows, Figure 10a,e–g,i,j and Figure 11a,d–f,i,j) was recorded. A peak in the maximum duration rate was observed during PII–PIII in the food-provision and center-diffusion areas (blue arrows, Figure 10a and Figure 11a), followed by a single peak in the maximum speed during P-S (green arrows, Figure 10b and Figure 11b). It can be conjectured that the individuals stayed longer in the resource supply areas during PIII due to feeding. Subsequently, the speed increased to a maximum during P-S as a result of this energy intake (Figure 5e).

A peak during PII–PIII was also observed for the dispersion parameters, MC and SSI, in the food-provision and center-diffusion areas (blue arrows, Figure 10i,j and Figure 11i,j), and this was associated with the cluster numbers in the food-provision area (blue arrow, Figure 10g). This suggested that local aggregation was maximized during PII–PIII before reaching a maximum speed during P-S. In fact, peaks for MC (Figure 10i and Figure 11i) and SSI (Figure 10j) were observed even earlier during PII than the peak cluster numbers during PIII (Figure 10g and Figure 11g), indicating that local crowdedness and balancing between attraction and repulsion with neighboring individuals started before the maximum clustering for feeding during PIII. Based on evidence from the *I*-index, individual isolation was also minimized during P-S (Figure 10h and Figure 11h) when speed was maximized in the food-provision and center-diffusion areas.

Trends of temporal co-occurrence were also observed in the areas of activity. In particular, the stop number and SSI were associated in the intermediate and edge areas across the light phases (green dotted rectangles, Figure 12e,j and Figure 13e,j). This indicated that the number of stops was concurrently associated with adjustments to the distance to maintain a balance between attraction and repulsion regarding neighboring individuals. These similarities for the SSI and stop number were also observed with the mutant strain *tab2^201Y^* (Figure 12e,j and Figure 13e,j), meaning that they were preserved even after genetic differentiation. Separately, the trend in cluster numbers was similar to that for speed in the center-diffusion and intermediate areas for Canton-S and *tab2^201Y^*.

In the scotoperiod, coinciding patterns between parameters were also observed. In particular, a continuous decrease after P-S was observed in the speed and the *I*-index in most areas, except the intermediate area (Figure 10, Figure 11 and Figure 13). In addition, decreases during SI followed by increases during SII were observed for the duration rate, stop number, stop time, MC, and SSI in the food-provision and center-diffusion areas (Figure 10 and Figure 11), indicating local aggregations to obtain resources along with maximum durations in the resource-supply areas.

These results suggest that spatial group behavior was illustrated effectively by the movement and dispersion parameters. The temporal co-occurrence patterns observed concurrently for the movement and dispersion parameters provide useful information on collective behavior mechanisms in response to external biological (e.g., neighbors) and environmental (e.g., food) factors. The underlying mechanisms responsible for spatial group formation in micro-areas as time progresses, however, are currently unknown. More research is required to investigate physiology–behavior relationships under a diverse range of experimental conditions.

Behavioral differences due to genetic differences were observed in the present study. Differences between wild-type strain Canton-S and mutant strain *tab2^201Y^* were recorded for various movement and dispersion parameters. A particularly notable difference was in the speed between the two strains (Figure 4a). While a peak was observed during P-S for Canton-S, the speed increased continuously after P-S for *tab2^201Y^*. This difference arose from the very high speeds observed for *tab2^201Y^* in the intermediate (Figure 12b) and edge (Figure 13b) areas, especially during the scotoperiod.

The mutant *tab2^201Y^*, as a mushroom body-specific GAL4 driver [42,43], has been used as a reference strain for investigating behavioral abnormalities after inducing the expression of genes under the control of UAS. It was originally assumed that *tab2^201Y^* would not exhibit major behavioral changes. Indeed, most studies have shown no significant alterations in complex behaviors including olfactory learning [62], and social behavior [29]. However, it was reported that the *tab2^201Y^* homozygous line affected courtship song [20]. In that study, the rhythm and pattern of the song pulses were significantly altered in the *tab2^201Y^* strain, leading to changes in the inter-pulse interval (IPI), which is the time between successive pulses in the song. Theses alterations in the courtship song can impact the mating success of males. Since the GAL4 trans-gene (p*{GawB}*) in the *tab2^201Y^* is inserted within the first intron of TAK1-assosicated binding protein 2 (*tab2*), which encodes a protein with a ubiquitin biding domain, these results suggested that *tab2* might have a role in courtship song [20]. However, molecular mechanisms underlying courtship song alterations in *tab2^201Y^* strain have not been unveiled. Similarly, the reasons for the differences in the group behavior observed for *tab2^201Y^* in the present study are currently unknown; thus, future neuro-physiological genetic research is required in order to understand these patterns.

Previous studies have investigated group behavior due to genetic differentiation using a variety of different methods. Schneider et al. [32] highlighted the benefits of integrating the history and pattern of interactions among individuals when identifying the molecular mechanisms that underlie the social modulation of behavior. Related research on physio-genetic mechanisms has also been reported, with the social space affected by parental age [63], genotype-by-social-environment interactions [12], and aggression due to social isolation [5]. In this respect, the present study provides a methodological foundation for investigating differences in behavior due to genetic composition, based on the use of micro-areas.

The proposed methods in this study could potentially be applied to understanding social behavior, adaptation, and evolution in *Drosophila*. Considering that collective behavior is focused on overall spatial conformation without specific interactions between individuals via visual and olfactory cues and without strictly requiring individual identification at the same time, group behaviors responding to internal stressors (e.g., starvation, aging) could be effectively elaborated along with behavior profiles and multiple parameter responses across different micro-areas. Although only male groups were observed in relatively low density in this study, both females and males could be further observed across different levels of group density in the observation arena to reveal a full scope of social behaviors in inter- and intra-sex relationships, including courtship, aggregation, and aggression.

Regarding adaptation and evolution, investigations of substrain differences in *Drosophila*, similarly conducted for locomotive behaviors [42], could be applied to group behaviors. Considering observation could be continuous for a long time, the proposed methods could be effectively used for revealing temporal acclimation and adaptation to changes in external conditions (e.g., temperature). Spatial conformation processes could be characterized comprehensively in multi-parameter dynamics in diverse aspects of local enhancement, including group formation (cluster numbers), local crowdedness (MC), social isolation (*I*-index), and attraction–repulsion balance (SSI) during the course of adaptation. This type of group behavior approach could be further utilized in evolution studies by investigating inter-generation heritability of local enhancement, like the case of heritability observed in the aggressive behavior of *Drosophila* [14].

Though behavioral differences were observed for groups in the present study, additional observations of the movement of individuals are required to determine whether these are qualitatively different from group behaviors or whether they vary between strains. However, given individual variability, a large number of trials would be required to observe individuals separately. Another future goal is to observe the same individual within a group by tracking every individual consistently over a long period of time.

Considerable variability in the parameters was observed between micro-areas and strains in the present study (e.g., Figure 14). However, even though the range of the SDs was high, the mean values for a number of the parameters were remarkably similar across micro-areas and between the two strains, such as the stop number and SSI in the intermediate and edge areas (Figure 12e,j and Figure 13e,j). These results contribute to making the parameter measurements reliable and support the understanding of collective behavior mechanisms by allowing crosschecking between parameters.

The CVs varied broadly in accordance with the micro-area, light phase, and strain (Figure 14). While sinuosity exhibited the broadest range of CVs in the food-provision and center-diffusion areas, the sessility parameters had the broadest range in the food-provision area. The range of CVs was also broader for *tab2^201Y^* than for Canton-S. In contrast, the DCR had the narrowest range of CVs across the micro-areas and strains. This variability can be used to investigate the degree of plasticity in group behavior. Indeed, behavioral variability related to genetic composition has been investigated for aggression [64], courtship [65], activity levels [66], grooming [67,68], and movement [69]. Genetic variation due to environment-constructing traits and social interactions have been reported to play causal roles in the plasticity of behavioral development in groups [16,70], in conjunction with physiological factors [13]. Our study constructed behavior profiles from the continuous observation of multiple parameters in different areas, and this would be an effective strategy for understanding plasticity in movement behaviors.

In addition to data variability, an extra property of measurement dependency was embedded in the spatio-temporal movement data in this study. Two points were considered for statistical differentiation in dependent measurements: finding statistical significances in the coupled dependence of micro-areas and light phases, and revealing specific differences among treatments within single factors (i.e., differences between micro-areas in each light phase and differences between light phases in each micro-area). Two-way repeated measurement ANOVA was conducted to analyze coupled dependence for the first point (see Section 2.4, *Statistical Analysis*). The test confirmed the existence of significant effects in micro-areas and light phases in the two strains (Table 1). For the second point, the Friedman, Wilcoxon signed-rank and paired permutation tests were conducted by releasing the coupled dependence to single dependence (see Section 2.4, *Statistical Analysis*). According to the Friedman test for evaluating total differences in the treatments of micro-areas and light phases separately, statistical significances were also observed in various combinations of micro-areas and light phases (Table 2 and Table 3), again confirming significant effects of the treatments in terms of single dependence. However, statistical significances were not observed for multiple comparisons among treatments between micro-areas and light phases, since the conservative criteria for alpha errors were applied to results obtained by Wilcoxon signed-rank and paired permutation tests. Probabilities for determining alpha errors were substantially decreased due to the extra degree of freedom caused by the number of comparisons (see Section 2.4, *Statistical Analysis*, Table 1, Table 2 and Table 3 and Figure 15 and Figure 16). To obtain statistical significance in multiple comparisons, more studies will be needed in the future including increase in trial numbers and decrease in the source of biological variability, for instance, specification of physiological status (e.g., age) across a narrow range of test individuals.

Although statistical significance was not observed in multiple comparisons, the trends of behavioral differences were illustrated according to micro-areas and light phases and were comparable between two strains (Figure 15 and Figure 16). Relative differences in parameters were more strongly observed for Canton-S than for *tab2^201Y^*. The differences in sinuosity were consistently statistically significant between micro-areas across the light phases, whereas the DCR was not statistically different (Figure 15). Among the micro-areas, significant differences in group behavior were more clearly observed in the intermediate area and for sessility parameters. The information provided in this study is useful for differentiating group behaviors based on genetic factors.

In this study, we opted to provide sugar for food instead of yeast, considering that simple minimal nutrition was suitable. To maintain the quality of food in long-term observations of behavioral responses, nutrients, the source of which are clearly identified, would be more suitable for the initial steps of collective behavior studies. For the next research steps, yeast and the various natural components within it should be used for observing group behavior and the results compared with those using food with minimal nutrients. In the present study, the duration rate in the edge area during PI was high compared with the other micro-areas, being coincident with high numbers of stops and time (Figure 13a). This may be because the starting location for the flies was the edge area. Although the flies were active after the acclimation period, they may have stayed longer in the edge area during the early photoperiod. Thus, more research is required to investigate group behavior in the edge area after using various forms of acclimation, including the use of anesthesia for medical purposes [71].

Automatic continuous monitoring of the group movements of *D. melanogaster* over 24 h using parameters based on instantaneous movement and dispersion of cumulated movement positions confirmed the natural tendency for local enhancement at a relatively low density. Temporal co-occurrence patterns in the measured parameters were observed over time in the areas providing resources, with a peak in the duration rates along with maximum local aggregation early during the photoperiod (PII–PIII), followed by a peak in the maximum speed along with the cluster numbers during the transition from the photoperiod to the scotoperiod (P-S). Other coinciding patterns were found in the areas related to activity between the stop number and SSI across the light phases. The group spacing was effectively represented by the movement and dispersion parameters, and the results provide in-depth information on the collective behavior mechanisms in response to biological and environmental factors. However, the specific factors responsible for spatial group formation in the micro-areas over time are currently unknown. More research is required to better understand physiology–genetics–behavior relationships under various experimental conditions.

Differences between the wild-type Canton-S and mutant *tab2^201Ỳ^* strains were observed in various movement and dispersion parameters, with a notably higher speed for *tab2^201Ỳ^* in the intermediate and the edge areas, especially during the scotoperiod. The current study thus provides a methodological foundation for monitoring group behavioral differences under various experimental conditions associated with different micro-areas.

## Figures and Tables

**Table 1:** Statistical differences in movement and dispersion parameters with dependent measurements in micro-areas and light phases within each trial (coupled dependence) according to two-way repeated ANOVA. (**a**) Within trials for Canton-S and *tab2^201Y^*, (**b**) between trials for Canton-S and *tab2^201Y^*.

(**a**)					
	**Parameters**	**Canton-S**		***tab2^201Y^***	
		***F***	***p***	***F***	***p***
Micro-area	Speed	18.081	0.000	3.570	0.130
	Locomotory rate	14.823	0.000	3.741	0.121
	DCR	1.085	0.349	1.026	0.398
	Sinuosity	49.595	0.000	20.280	0.009
	Stop numbers	20.880	0.001	19.156	0.000
	Stop time	21.560	0.000	8.289	0.027
	Cluster numbers	21.098	0.002	7.335	0.047
	*I*-index	4.611	0.073	6.384	0.064
	MC	3.247	0.081	7.111	0.017
	SSI	19.411	0.000	4.809	0.043
Light phase	Speed	3.849	0.044	1.822	0.234
	Locomotory rate	3.702	0.049	1.832	0.234
	DCR	1.846	0.185	0.077	0.904
	Sinuosity	1.072	0.371	0.621	0.485
	Stop numbers	7.812	0.002	1.094	0.380
	Stop time	2.746	0.083	0.259	0.728
	Cluster numbers	2.338	0.131	3.094	0.127
	*I*-index	1.278	0.314	0.909	0.438
	MC	2.254	0.135	1.219	0.345
	SSI	4.643	0.040	1.194	0.347
Micro-area × Light phase	Speed	3.005	0.061	1.730	0.242
	Locomotory rate	2.960	0.066	1.443	0.295
	DCR	1.257	0.320	1.360	0.310
	Sinuosity	1.172	0.344	0.667	0.496
	Stop numbers	8.314	0.000	1.852	0.208
	Stop time	5.720	0.004	1.909	0.208
	Cluster numbers	4.258	0.025	1.929	0.222
	*I*-index	1.442	0.277	0.614	0.582
	MC	1.724	0.196	3.108	0.106
	SSI	6.865	0.006	1.378	0.308
(**b**)					
	**Parameters**	**Canton-S**		***tab2^201Y^***	
		***F***	***p***	***F***	***p***
	Speed	107.083	0.000	9.199	0.039
	Locomotory rate	122.142	0.000	10.330	0.032
	DCR	1497.191	0.000	2190.677	0.000
	Sinuosity	538.591	0.000	137.749	0.000
	Stop numbers	38.702	0.001	71.848	0.001
	Stop time	102.805	0.000	29.834	0.005
	Cluster numbers	390.028	0.000	34.013	0.004
	*I*-index	514.193	0.000	273.386	0.000
	MC	84.686	0.000	12.144	0.025
	SSI	253.027	0.000	24.261	0.008

**Table 2:** Statistical differences in movement and dispersion parameters with dependent measurements of light phases in separate data sets for micro-areas, according to the Friedman test: (**a**) Canton-S and (**b**) *tab2^201Y^* (*χ*^2^ indicating the chi-square statistic for the Friedman test).

(**a**)												
Parameters	PI		PII		PIII		P-S		SI		SII	
	*χ* ^2^	*p*	*χ* ^2^	*p*	*χ* ^2^	*p*	*χ* ^2^	*p*	*χ* ^2^	*p*	*χ* ^2^	*P*
Speed	3.514	0.319	0.150	0.985	25.714	0.000	6.750	0.080	10.950	0.012	17.550	0.001
Locomotory rate	3.514	0.319	3.150	0.369	8.550	0.036	7.350	0.062	9.900	0.019	13.350	0.004
DCR	0.257	0.968	3.150	0.369	4.650	0.199	2.250	0.522	3.750	0.290	3.450	0.327
Sinuosity	14.829	0.002	20.250	0.000	22.200	0.000	15.450	0.001	19.500	0.000	22.950	0.000
Stop numbers	18.943	0.000	15.750	0.001	8.700	0.034	9.000	0.029	14.550	0.002	9.150	0.027
Stop time	18.943	0.000	11.850	0.008	8.250	0.041	10.950	0.012	15.450	0.001	9.450	0.024
Cluster numbers	19.971	0.000	18.600	0.000	17.250	0.001	14.550	0.002	19.950	0.000	19.050	0.000
*I*-index	15.343	0.002	19.950	0.000	8.550	0.036	5.250	0.154	12.150	0.007	11.550	0.009
MC	12.600	0.006	6.450	0.092	17.250	0.001	14.550	0.002	19.950	0.000	19.050	0.000
SSI	14.829	0.002	15.450	0.001	3.450	0.327	4.650	0.199	7.800	0.050	6.450	0.092
(**b**)												
Parameters	PI		PII		PIII		P-S		SI		SII	
	*χ* ^2^	*p*	*χ* ^2^	*p*	*χ* ^2^	*p*	*χ* ^2^	*p*	*χ* ^2^	*p*	*χ* ^2^	*P*
Speed	0.600	0.896	3.000	0.392	1.800	0.615	7.950	0.047	8.657	0.034	14.657	0.002
Locomotory rate	0.200	0.978	3.857	0.277	1.650	0.648	7.950	0.047	9.000	0.029	14.486	0.002
DCR	5.600	0.133	2.829	0.419	1.050	0.789	6.750	0.080	6.257	0.100	5.229	0.156
Sinuosity	12.200	0.007	15.343	0.002	21.600	0.000	16.350	0.001	17.914	0.000	14.486	0.002
Stop numbers	12.600	0.006	6.257	0.100	7.350	0.062	9.450	0.024	10.371	0.016	11.057	0.011
Stop time	12.200	0.007	6.600	0.086	8.850	0.031	9.450	0.024	10.029	0.018	10.543	0.014
Cluster numbers	14.600	0.002	13.800	0.003	14.550	0.002	12.600	0.006	13.971	0.003	16.200	0.001
*I*-index	11.160	0.011	14.600	0.002	11.600	0.009	12.200	0.007	13.457	0.004	14.143	0.003
MC	12.600	0.006	5.914	0.116	4.050	0.256	12.450	0.006	5.914	0.116	14.486	0.002
SSI	12.200	0.007	5.229	0.156	10.050	0.018	8.250	0.041	2.314	0.510	4.371	0.224

**Table 3:** Statistical differences in movement parameters and dispersion parameters with dependent measurements of light phases in separate data sets for micro-areas, according to the Friedman test, (**a**) Canton-S and (**b**) *tab2^201Y^* (*χ*^2^ indicating the chi-square statistic for the Friedman test).

(**a**)								
Parameters	Food-provision		Center-diffusion		Intermediate		Edge	
	*χ* ^2^	*p*	*χ* ^2^	*p*	*χ* ^2^	*p*	*χ* ^2^	*p*
Speed	12.878	0.025	19.000	0.002	25.714	0.000	16.500	0.006
Locomotory rate	10.673	0.058	16.000	0.007	24.714	0.000	15.929	0.007
DCR	12.959	0.024	2.714	0.744	12.714	0.026	6.786	0.237
Sinuosity	16.388	0.006	15.643	0.008	3.909	0.563	4.571	0.470
Stop numbers	9.857	0.079	24.214	0.000	9.071	0.106	21.500	0.001
Stop time	14.429	0.013	20.714	0.001	5.643	0.343	21.429	0.001
Cluster numbers	5.776	0.329	27.929	0.000	18.357	0.003	12.000	0.035
*I*-index	8.061	0.153	11.714	0.039	9.929	0.077	20.929	0.001
MC	3.490	0.625	13.143	0.022	4.429	0.489	14.571	0.012
SSI	4.959	0.421	11.571	0.041	5.429	0.366	20.786	0.001
(**b**)								
Parameters	Food-provision		Center-diffusion		Intermediate		Edge	
	*χ* ^2^	*p*	*χ* ^2^	*p*	*χ* ^2^	*p*	*χ* ^2^	*p*
Speed	0.886	0.971	3.245	0.662	17.204	0.004	20.959	0.001
Locomotory rate	2.143	0.829	4.143	0.529	16.061	0.007	21.939	0.001
DCR	1.686	0.891	4.551	0.473	3.571	0.613	2.918	0.713
Sinuosity	3.400	0.639	6.952	0.224	2.969	0.705	1.449	0.919
Stop numbers	3.171	0.674	11.816	0.037	8.714	0.121	11.327	0.045
Stop time	3.629	0.604	12.959	0.024	3.898	0.564	4.959	0.421
Cluster numbers	7.400	0.193	18.952	0.002	11.980	0.035	11.490	0.042
*I*-index	13.000	0.023	1.550	0.907	5.400	0.369	15.000	0.010
MC	4.429	0.489	7.524	0.185	11.245	0.047	17.857	0.003
SSI	1.800	0.876	5.048	0.410	8.878	0.114	7.408	0.192

**Table 4:** Statistical significances of movement and dispersion parameters between two strains in different light phases across micro-areas according to the Wilcoxon signed-rank and paired permutation tests (*W*, Δ*μ* indicating statistics based on median rank for the Wilcoxon signed-rank test and mean difference for the paired permutation test, respectively) (Blue color: *p* < 0.05; Green color: 0.05 ≤ *p* < 0.10).

Parameters and Phase		Total				Food-Provision				Center-Diffusion				Intermediate				Edge			
		Wilcoxon Signed-Rank		Paired Permutation		Wilcoxon Signed-Rank		Paired Permutation		Wilcoxon Signed-Rank		Paired Permutation		Wilcoxon Signed-Rank		Paired Permutation		Wilcoxon Signed-Rank		Paired Permutation	
		*W*	*p*	Δ*μ*	*p*	*W*	*p*	Δ*μ*	*p*	*W*	*p*	Δ*μ*	*p*	*W*	*p*	Δ*μ*	*p*	*W*	*p*	Δ*μ*	*p*
Speed	PI	4	0.055	0.235	0.039	1	0.063	0.323	0.031	4	0.055	0.252	0.008	5	0.078	0.915	0.257	1	0.016	0.232	0.023
	SII	2	0.047	1.005	0.062	9	0.469	0.218	0.155	10	0.578	0.154	0.264	3	0.078	1.546	0.186	1	0.031	1.684	0.047
Loco. rate	PI	1	0.016	0.347	0.016	0	0.031	0.411	0.031	4	0.055	0.265	0.086	5	0.078	1.072	0.226	0	0.008	0.337	0.031
	PII	16	0.844	0.158	0.459	13	0.938	0.023	0.837	11	0.383	−0.088	0.475	9	0.25	1.05	0.093	13	0.547	0.341	0.389
	SI	9	0.469	0.638	0.403	13	0.938	−0.023	0.992	14	1	0.036	0.961	1	0.031	1.782	0.295	6	0.219	1.217	0.217
	SII	3	0.078	1.124	0.031	10	0.578	0.206	0.202	6	0.219	0.206	0.264	2	0.047	1.762	0.031	2	0.047	1.924	0.047
DCR	PII	17	0.945	−1.536	0.879	3	0.078	13.555	0.31	17	0.945	4.145	0.825	14	0.641	0.585	0.981	17	0.945	−2.143	0.747
Sinu.	PI	14	0.641	106.77	0.195	6	0.438	3.03	0.308	1	0.031	10.474	0.047	17	0.945	23.401	0.607	12	0.461	−36.029	0.529
	SII	3	0.465	46.907	0.543	11	0.688	−2.325	0.465	12	0.813	−6.328	0.605	1	0.08	4.146	0.093	9	0.469	34.443	0.636
St. no.	PI	4	0.055	−331.312	0.008	8	0.688	7.78	0.554	14	0.641	17.904	0.467	7	0.148	−80.45	0.265	7	0.148	−161.837	0.109
	PII	3	0.039	−293.475	0.023	10	0.578	61.071	0.45	10	0.313	49.982	0.428	6	0.109	−69.875	0.311	12	0.461	−76.284	0.467
	PIII	7	0.148	−213.538	0.093	12	0.461	48.604	0.412	13	0.547	−22.475	0.732	6	0.109	−118.9	0.156	17	0.945	23.916	0.661
	P-S	10	0.313	−206.312	0.156	17	0.945	7.121	0.864	9	0.25	53.563	0.374	3	0.039	−150.037	0.031	17	0.945	38.375	0.7
St. ti.	P-S	11	0.383	−294.675	0.716	16	0.844	60.137	0.693	7	0.148	252.129	0.265	0	0.008	−360.7	0.125	18	1	8.787	0.996
Cluster	PIII	3	0.039	−2.973	0.101	18	1	0.029	0.84	12	0.461	−0.289	0.374	0	0.008	−2.878	0.016	17	0.945	0.527	0.654
	P-S	5	0.078	−4.033	0.163	17	0.945	0.015	0.786	3	0.039	−0.791	0.016	0	0.008	−4.847	0.016	16	0.844	1.601	0.459
	SI	11	0.688	−0.272	0.822	10	0.578	0.043	0.279	11	0.688	0.257	0.496	4	0.109	−3.162	0.093	5	0.156	4.134	0.186
	SII	8	0.375	3.488	0.403	14	1	−0.026	0.791	8	0.375	0.178	0.667	5	0.156	−1.205	0.124	1	0.031	5.71	0.031
*I*-Index	P-S	9	0.25	−0.016	0.342	1	0.016	−0.07	0.117	3	0.039	−0.063	0.062	13	0.547	0.294	0.265	5	0.078	−0.058	0.054
	SI	14	1	0.001	0.884	8	0.375	−0.018	0.589	12	0.813	−0.011	0.605	0	0.016	0.397	0.016	13	0.938	−0.022	0.527
	SII	12	0.813	0.007	0.605	13	0.938	0.011	0.636	8	0.375	0.024	0.295	4	0.109	0.333	0.047	11	0.688	0.015	0.791
MC	P-S	18	1	-4.991	0.591	17	0.945	0.124	0.911	10	0.313	9.093	0.226	7	0.148	-10.082	0.039	12	0.461	-3.101	0.381
	SII	10	0.578	-4.798	0.605	5	0.156	-6.693	0.233	10	0.578	7.454	0.357	4	0.109	-10.903	0.078	12	0.813	0.326	0.93
SSI	PI	4	0.055	−3315.56	0.031	9	0.844	437.05	0.738	12	0.813	−30.136	0.868	12	0.461	−478.324	0.584	2	0.023	−2711.53	0.047
	PII	7	0.148	−3128.22	0.062	12	0.813	227.655	0.977	7	0.148	−340.694	0.241	7	0.148	−567.741	0.179	3	0.039	−2507.21	0.078
	PIII	7	0.148	−1424.97	0.21	17	0.945	324.219	0.802	6	0.109	−737.317	0.078	6	0.109	−1102.44	0.039	14	0.641	−274.226	0.77
	P-S	17	0.945	76.322	0.996	18	1	319.303	0.833	12	0.461	171.639	0.553	8	0.195	−855.893	0.07	15	0.742	−317.488	0.506
	SII	12	0.813	221.863	0.884	11	0.688	−606.862	0.186	11	0.688	902.952	0.465	5	0.156	−1109.96	0.093	13	0.938	−167.561	0.853

## Data Availability

Software and data are available from the authors upon request.

## Abbreviations

The following abbreviations are used in this manuscript:

MC	Mean crowding
SSI	Social space index
SIN	Social interaction network
UAS	Upstream activating sequence
DCR	Direction change rate
CV	Coefficient of variation
Inter.	Intermediate
Loco. Rate	Locomotory rate
Sinu.	Sinuosity
St. no.	Stop number
St. ti.	Stop time
